# Multi-omics analysis of human tendon adhesion reveals that ACKR1-regulated macrophage migration is involved in regeneration

**DOI:** 10.1038/s41413-024-00324-w

**Published:** 2024-05-07

**Authors:** Xinshu Zhang, Yao Xiao, Bo Hu, Yanhao Li, Shaoyang Zhang, Jian Tian, Shuo Wang, Zaijin Tao, Xinqi Zeng, Ning-Ning Liu, Baojie Li, Shen Liu

**Affiliations:** 1 Department of Orthopaedics, Shanghai Sixth People’s Hospital Affiliated to Shanghai Jiao Tong University School of Medicine, 600 Yishan Rd, Shanghai, 200233 PR China; 2 Section of Spine Surgery, Department of Orthopaedics, Changzheng Hospital, Naval Medical University, Shanghai, 200003 PR China; 3 Bio-X Institutes, Key Laboratory for the Genetics of Developmental and Neuropsychiatric Disorders, Ministry of Education, Shanghai Jiao Tong University, Shanghai, 200241 PR China; 4 Department of Orthopaedics, Wuxi Ninth People’s Hospital Affiliated to Soochow University, Wuxi, 214062 PR China; 5 State Key Laboratory of Systems Medicine for Cancer, Center for Single-Cell Omics, School of Public Health, Shanghai Jiao Tong University School of Medicine, Shanghai, 200025 China

**Keywords:** Pathogenesis, Diseases

## Abstract

Tendon adhesion is a common complication after tendon injury with the development of accumulated fibrotic tissues without effective anti-fibrotic therapies, resulting in severe disability. Macrophages are widely recognized as a fibrotic trigger during peritendinous adhesion formation. However, different clusters of macrophages have various functions and receive multiple regulation, which are both still unknown. In our current study, multi-omics analysis including single-cell RNA sequencing and proteomics was performed on both human and mouse tendon adhesion tissue at different stages after tendon injury. The transcriptomes of over 74 000 human single cells were profiled. As results, we found that SPP1^+^ macrophages, RGCC^+^ endothelial cells, ACKR1^+^ endothelial cells and ADAM12^+^ fibroblasts participated in tendon adhesion formation. Interestingly, despite specific fibrotic clusters in tendon adhesion, FOLR2^+^ macrophages were identified as an antifibrotic cluster by in vitro experiments using human cells. Furthermore, ACKR1 was verified to regulate FOLR2^+^ macrophages migration at the injured peritendinous site by transplantation of bone marrow from *Lysm-Cre;R26R*^*tdTomato*^ mice to lethally irradiated *Ackr1*^*−/−*^ mice (*Ackr1*^*−/−*^ chimeras; deficient in ACKR1) and control mice (WT chimeras). Compared with WT chimeras, the decline of FOLR2^+^ macrophages was also observed, indicating that ACKR1 was specifically involved in FOLR2^+^ macrophages migration. Taken together, our study not only characterized the fibrosis microenvironment landscape of tendon adhesion by multi-omics analysis, but also uncovered a novel antifibrotic cluster of macrophages and their origin. These results provide potential therapeutic targets against human tendon adhesion.

## Introduction

Tendon adhesion is a common complication after tendon injury that limits limb mobility with a growing concern affecting approximately 33.2 injuries per 100 000 person-years in America and causing injuries in 2.5 million people globally each year, results from a series of tendon injuries that progressively cause fibrosis.^1,2^ The recurrence of tendon adhesion after tenolysis is known to be directly related to unfavorable patient outcomes, emphasizing the pressing need for effective anti-fibrotic therapies. However, the mechanism remains unknown, which limits the improvement of treatment in clinic.

During the process of tendon healing after surgical repair, three overlapping phases are commonly recognized inflammation (days 1–7), proliferation (days 3–14), and remodeling (day 10 onward). Along with the tendon healing, the peritendinous adhesion is formed by excessive invasion of granulation tissue from surrounding tissues.^3,4^ Recently, the well-known intricate process of tendon adhesion formation is driven by the interaction among various peritendinous cell lineages, encompassing immune, endothelial, and mesenchymal cells.^5^ These cells are found within specialized fibrotic areas, referred to as the fibrotic niche. Although rodent models provide a chance to investigate the advanced understanding of peritendinous fibrogenesis, a considerable challenge still persists between the identification of potential therapeutic targets and the development of successful treatments.^6,7^ This is partly due to insufficient characterization of the functional diversity and interactions among cell lineages involved in the human tendon adhesion fibrotic niche, which cannot be entirely replicated in rodent models.^6^

The widely reported tendon adhesion related cells are macrophages.^7^ The key function of macrophages is considered as a fibrotic trigger to regulate the fibroblasts and myofibroblasts.^8,9^ Based on our previous study, macrophages were found to secret TGFβ1 and subsequently recruit stem cell-derived myofibroblasts for adhesion formation.^10^ However, their exact cluster and origin remain unknown. To solve this problem, we profiled 74 350 cells from human tendon adhesion tissue at three time points following tendon injury using multi-omics analysis including single-cell RNA sequencing (scRNA-seq) and proteomics to study the interactions between different cell lineages. To verify the function of determined clusters, specific macrophages cluster was sorted and in vitro investigated. In addition, transplantation of bone marrow from lineage-tracing mice, inducible Cre mouse models and global knockout mice were used to verify the relationship between the antifibrotic cluster and endothelial cells. These results uncover potential therapeutic targets against human tendon adhesion.

## Results

### Single-cell transcriptome atlas of human tendon adhesion tissue

**Fig. 1:**
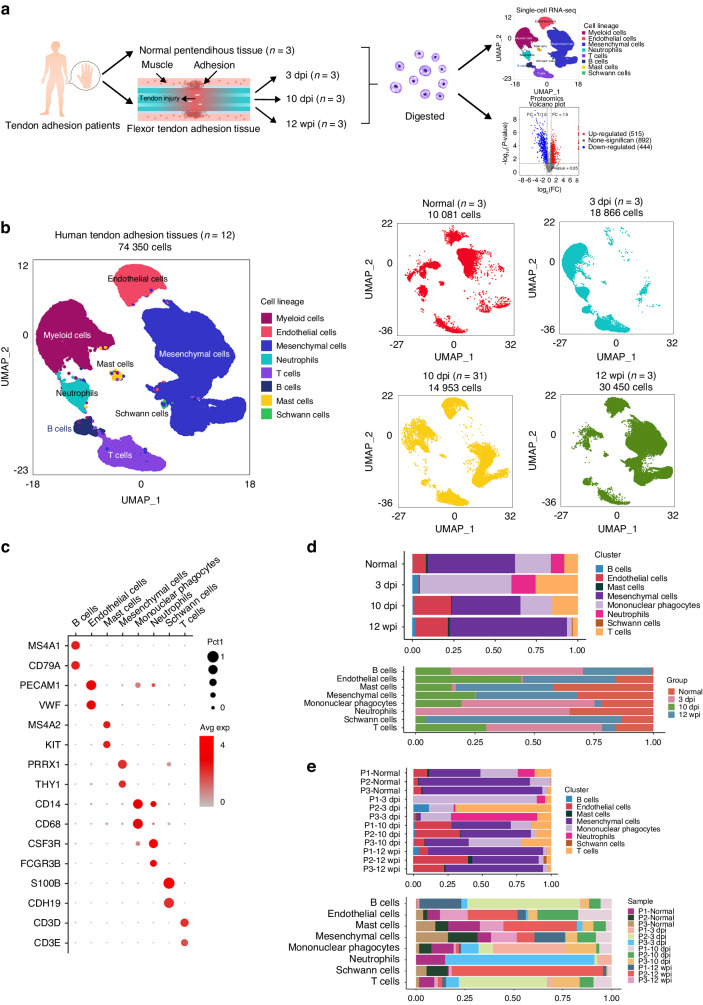
Single-cell atlas of human normal peritendinous tissue and tendon adhesion tissue. **a** Overview of this study design. Normal peritendinous tissue and tendon adhesion tissue were collected from tendon injury patients and processed for 3′-end scRNA-seq using the 10× Genomics platform. **b** UMAP plots of 74 350 cells from total 12 patients with tendon injury. 10 081 cells from normal peritendinous tissue of three patients. 18 866 cells from tendon adhesion tissue of three 3 dpi (day post injury) patients. 14 953 cells from tendon adhesion tissue of three 10 dpi patients. 30 450 cells from tendon adhesion tissue of three 12 wpi (week post injury) patients. **c** Dot plot: showing cell clusters of human tendon adhesion tissue by known markers. The dot size indicates the gene expression percent in each cluster. The color indicates mean gene expression (Red, high). **d** Bar plots of the proportion of eight major cell types in human tendon adhesion tissue of each time point. **e** Bar plots of the proportion of eight major cell types in human tendon adhesion tissue of each patient

To determine the cellular composition of tendon adhesion tissue, tissue samples were obtained surgically from 12 patients with flexor tendon tenolysis including three samples of normal peritendinous tissue and three pathologic samples from the lesion at 3, 10 days post injury (dpi) and 12 weeks post injury (wpi), respectively. The samples were immediately processed for 3′-end scRNA-seq using the 10× Genomics platform (Fig. 1a and Table 1). The data were clustered and integrated by time point, and clusters that were of low quality or were doublets were removed. A total of 74 350 cell transcriptomes from the 12 patients were retained for subsequent analysis, of which 10 081 cells originated from normal peritendinous tissue, 18 866 from 3 dpi tissue, 14 953 from 10 dpi tissue, and 30 450 from 12 wpi tissue (Fig. 1b and Table 2).

**Table 1:** Clinical characteristics of 12 patients in the study

Sample ID	Pationt ID	Age/year	Sex	Flexor tendon lesion	Tendon injury time
1	P1-Normal	64	male	thumb	/
2	P2-Normal	31	male	index finger	/
3	P3-Normal	52	female	thumb	/
4	P1-3 dpi	71	female	ring finger	2.7 days
5	P2-3 dpi	28	male	thumb	3.5 days
6	P3-3 dpi	37	female	index finger	3.3 days
7	P1-10 dpi	25	maie	middle finger	9.1 days
8	P2-10 dpi	49	male	thumb	11.2 days
9	P3-10 dpi	61	female	thumb	10.7 days
10	P1-12 wpi	53	female	index finger	12.5 weeks
11	P2-12 wpi	39	male	ring finger	11.6 weeks
12	P3-12 wpi	45	male	index finger	12.3 weeks

**Table 2:** Statistics of scRNA-seq

Sample ID	Patient ID	Number of Cells	Mean Reads per Cell	Median Genes per Cell
1	P1-Normal	6 646	51 611	1 622
2	P2-Normal	2 072	121 410	3 279
3	P3-Normal	1 363	134 340	1 829
4	P1-3 dpi	9 711	30 382	1 940
5	P2-3 dpi	5 786	44 468	1 417
6	P3-3 dpi	3 369	121 764	737
7	P1-10 dpi	5 717	48 267	2 624
8	P2-10 dpi	4 510	61 737	2 685
9	P3-10 dpi	4 726	52 813	2 529
10	P1-12 wpi	13 585	23 270	1 637
11	P2-12 wpi	7 376	30 982	1 567
12	P3-12 wpi	9 489	31 870	1 414

An atlas of cell types in normal peritendinous and injured tissue was established by first clustering nuclei at a coarse level and then annotating each cluster with cell type-specific markers (Fig. 1c). We identified eight major cell clusters including mononuclear phagocytes (MPs, *n* = 16 299) identified by CD14 molecule (*CD14)* and CD68 molecule (*CD68)*^11,12^ (Fig. S1B), endothelial cells (ECs, *n* = 10 175) marked by platelet and endothelial cell adhesion molecule 1 (*PECAM1)* and von Willebrand factor (*VWF)*^13,14^ (Fig. S1B), mesenchymal cells (MCs, *n* = 33 284) identified by paired related homeobox 1 (*PRRX1)*^15^ and Thy-1 cell surface antigen (*THY1)*^16^ respectively (Fig. S1C), neutrophils (*n* = 3 578) marked by colony stimulating factor 3 receptor (*CSF3R)* and Fc gamma receptor IIIb (*FCGR3B)*^17^ (Fig. S1D), B cells (*n* = 1 425) expressed membrane spanning 4-domains A1 (*MS4A1)* and CD79a molecule (*CD79A)*^18^ (Fig. S1E), T cells (*n* = 8 914) expressed CD3 delta subunit of T-cell receptor complex (*CD3D)* and CD3 epsilon subunit of T-cell receptor complex (*CD3E)*^19^ (Fig. S1F), mast cells (*n* = 497) marked membrane spanning 4-domains A2 (*MS4A2)* and KIT proto-oncogene, receptor tyrosine kinase *(KIT)*^20^ and schwann cells (*n* = 178) identified by cadherin 19 (*CDH19)* and S100 calcium binding protein B (*S100B)*^21^ (Fig. S1G, H; and Table 3).

**Table 3:** Cell composition of 12 samples

Patient ID	Mononuclear phagocytes	Endothelial cells	Mesenchymal cells	B cells	T cells	Neutrophils	Mast cells	Schwann cells
P1-Normal	1 808	664	2 472	3	802	808	88	1
P2-Normal	314	67	1 674	/	/	/	11	6
P3-Normal	53	78	1 185	1	33	/	11	2
P1-3 dpi	8 665	/	/	21	444	581	/	/
P2-3 dpi	1 030	/	/	652	4 032	72	/	/
P3-3 dpi	729	59	92	20	344	2 117	8	/
P1-10 dpi	859	1 522	2 443	62	814	/	14	3
P2-10 dpi	166	1 473	2 300	52	496	/	23	/
P3-10 dpi	1 787	357	1 501	32	1 026	/	22	1
P1-12 wpi	355	918	11 332	559	414	/	1	6
P2-12 wpi	257	2 927	3 562	7	242	/	226	155
P3-12 wpi	276	2 110	6 723	16	267	/	93	4
Total	16 299	10 175	33 284	1 425	8 914	3 578	497	178

### Characteristics of cell populations in tendon adhesion tissue of different clinical stage

The proportion of inflammatory cells, ECs, and MCs varied according to the stage of tendon adhesion (Fig. 1d, e). In the normal peritendinous tissue, MCs were the most abundant cell type, accounting for 52.9% of the total (Fig. 1d and Table 4). At 3 dpi (inflammatory phase)^3^, the proportion of MCs and ECs was lower (0.3% and 0.5% of total cells, respectively), whereas the proportion of inflammatory cells including mononuclear phagocytes, T cells, B cells and neutrophils increased; the most abundant cell type at this stage was MPs (55.3%), followed by T cells (25.5%) and neutrophils (14.7%) (Fig. 1d and Table 4). At 10 dpi (proliferative phase)^3^, the number of inflammatory cells had declined, with macrophages accounting for just 18.8% of all cells. Meanwhile, ECs and MCs had undergone extensive proliferation and accounted for 22.4% and 41.8% of cells, respectively (Fig. 1d and Table 4). At 12 wpi (remodeling phase)^3^, fibroblasts had migrated to the lesion and were present in the granulation tissue, which induces tendon adhesion. MCs (71%) and ECs (19.6%) were the major cell types at this stage, with few inflammatory cells (Fig. 1d and Table 4). The profile of the major cell types at 12 wpi was similar to that of normal peritendinous tissue, indicating that at an advanced stage of tendon adhesion, the tissue state was stabilizing. Based on these dynamics of cell populations, we speculated that MPs, ECs, and MCs play an important role in the development of tendon adhesion.

**Table 4:** Cell composition of total samples at different stages

Sample ID	Mononuclear phagocytes/%	Endothelial cells/%	Mesenchymal cells/%	B cells/%	T cells/%	Neutrophils/%	Mast cells/%
Total normal	21.5	8	52.9	0	8.3	8	0
Total 3 dpi	55.3	0.3	0.5	3.7	25.5	14.7	0
Total 10 dpi	18.8	22.4	41.8	1	15.6	0	0.4
Total 12 wpi	2.9	19.6	71	1.9	3	0	1.1

### *ADAM12* expression defines multipotent profibrogenic mesenchymal stromal cells

In human tendon adhesion tissue, mesenchymal cells (MCs) were categorized into four subsets based on distinct markers: ADAM metallopeptidase domain 12 (*ADAM12)*, C-X-C motif chemokine ligand 14 (*CXCL14)*, regulator of G protein signaling 5 *(RGS5)* and myosin heavy chain 11 (*MYH11)* (Fig. S2A-D). Two samples at 3 dpi had no MCs, as they were scarce at this time point (Fig. S2F and Table 3). *ADAM12* and periostin (*POSTN)* was the marker of MC0 and identified as fibroblasts (Fig. S2B, C). MC1 was distinguished by *CXCL14* expression, was identified as fibroblasts (Fig. S2B, C). MC2 expressed high levels of *RGS5*, STEAP4 metalloreductase (*STEAP4)* and collagen type IV alpha 1 chain (*COL4A1)* was identified as pericytes^22^ (Fig. S2B). MC3 was marked by *MYH11* and actin alpha 2, smooth muscle (*ACTA2)* and identified as myofibroblasts^23^ (Fig. S2B, C). The proportions of the four subsets revealed that MC1 primarily constituted MCs in normal peritendinous tissue (Fig. S2E). MC0 and MC2 proliferated in 10 dpi tissue, while MC1 and MC3 proliferated in 12 wpi tissue (Fig. S2E). After tendon injury, a total of three stages compared with normal peritendinous tissue, the upregulated signaling pathways of MCs in tendon adhesion tissue including ECM-receptor interaction and focal adhesion (Fig. S2G). Visualization of the pseudotemporal trajectory and RNA velocity suggested that MC0 differentiated into MC1, followed by MC1 differentiating into MC2, and ultimately MC2 differentiating into MC3 after tendon injury, 3 dpi to 10 dpi then to 12 wpi (Fig. S3A and Fig. S4A–C), and the origin of MC0 was bone-marrow. This observation implies that tendon injury may induce pericyte-myofibroblast transition (PMT) during human tendon adhesion. MCs in human normal peritendinous tissue were primarily CXCL14^+^ fibroblasts (Fig. S2E).

ADAM12^+^ cells are known progenitors of a large proportion of collagen-overproducing cells generated during scarring that are progressively eliminated during healing.^24^ MC0 expressed high levels of profibrotic genes including *COL1A1*, collagen type III alpha 1 chain (*COL3A1)* and *POSTN*^25^ (Fig. S2C). The Gene Ontology enrichment analysis suggested MC0 participated in collagen fibril organization and ECM organization (Fig. S3B), and the QuSAGE analysis of enriched pathways showed that genes related to ECM–receptor interaction and platelet activation that are associated with fibrosis were upregulated in MC0^26–28^ (Fig. S3C). The gene enrichment of MC0 ECM-receptor interaction hallmark gene set suggested the profibrotic genes including collagen type I alpha 2 chain (*COL1A2)* and *FN1* upregulated (Fig. S4D), which was supported by KEGG pathway analysis (Fig. S3D). The heatmap of TFs revealed that MC0 were enriched in TFs that promote fibrosis including hypoxia-inducible factor 1 subunit alpha (*HIF1A)*^29^ (Fig. S4E). To further investigate MC0, we isolated ADAM12^+^ MCs from 10 dpi human adhesion tissue and induced them to undergo adipogenic, osteogenic, and chondrogenic differentiation (Fig. S4F). The results indicated that ADAM12^+^ fibroblasts were multipotent stromal cells. Immunofluorescence demonstrated colocalization of *ADAM12* and *CXCL14* around collagen, as well as colocalization of COL4A1^+^ pericytes with MYH11^+^ myofibroblasts in 10 dpi tissue (Figs. S3E and S4G). These findings indicate that in the progression of human tendon adhesion, ADAM12^+^ multipotent stromal cells might transform into CXCL14^+^ fibroblasts, while COL4A1^+^ pericytes could convert into MYH11^+^ myofibroblasts. PMT could play a role in human tendon adhesion.

In summary, ADAM12^+^ cells, as profibrotic multipotent stromal cells, proliferate during the intermediate stages of tendon adhesion after injury and differentiate into CXCL14^+^ fibroblasts, possibly playing a vital part in this process. Moreover, PMT might be crucial for human tendon adhesion.

### Unique subgroups of endothelial cells reside within the fibrotic microenvironment

**Fig. 2:**
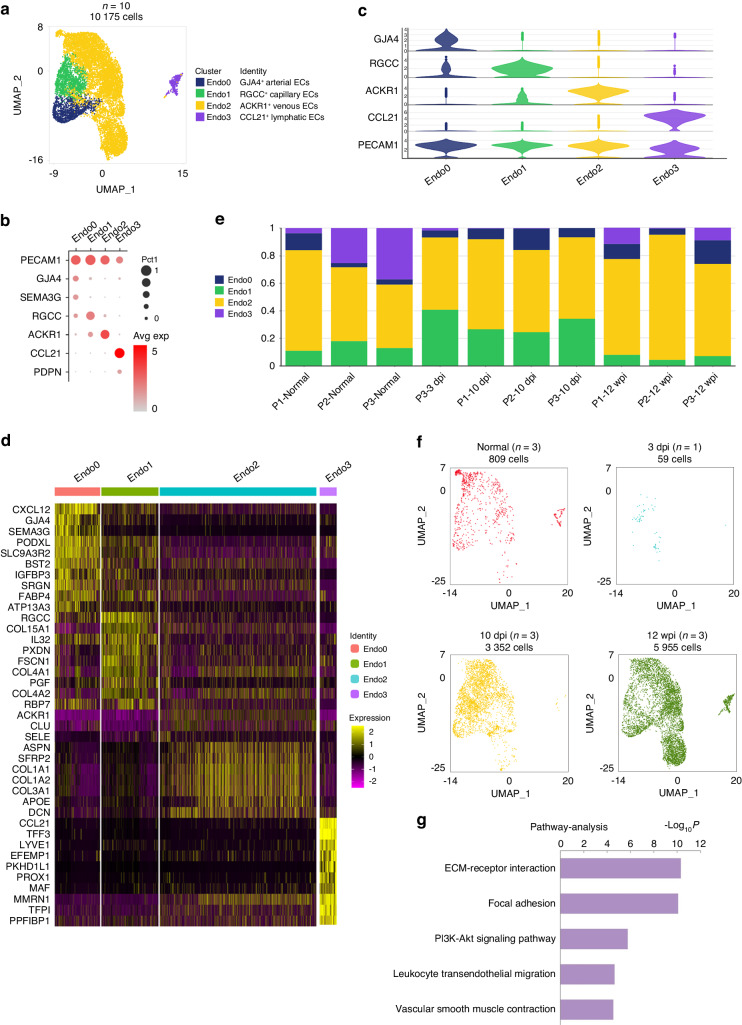
Distinct EC clusters present in human tendon adhesion tissue. **a** Clustering 10 175 endothelial cells from total 10 patients. ECs, endothelial cells. **b** Dot plot: showing cell clusters of endothelial cells by known markers. The dot size indicates the gene expression percent in each cluster. The color indicates mean gene expression (Red, high). **c** The violin plot of selected gene expression of each cluster in ECs. **d** Heatmap of marker genes in each EC cluster. Top, clusters. Left, marker genes. **e** Bar plots of proportion of each cluster in ECs from 10 patients. **f** UMAP plots of ECs of each time point: normal, 3 dpi, 10 dpi and 12 wpi. **g** Pathway analysis of upregulated signaling pathway of all three stages after tendon injury

Endothelial cells (ECs) in tendon adhesion tissue were identified by the high expression levels of *PECAM1* and *VWF*^13,14^ (Fig. 1c). Then the ECs were further subclustered into four subpopulations based on different markers including gap junction protein alpha 4 (*GJA4)*, regulator of cell cycle (*RGCC)*, atypical chemokine receptor 1 (*ACKR1)* and C-C motif chemokine ligand 21 (*CCL21)* (Fig. 2a–d). ECs were scarcely present in human tendon adhesion tissue at 3 dpi, with two samples lacking ECs (Fig. 2e, f and Table 3). *GJA4* and semaphorin 3G (*SEMA3G)* were specially expressed in human arteries, suggesting ENDO0 as arterial ECs^14,30^ (Fig. 2b, c). *RGCC* was reported as the marker of human capillary which suggested ENDO1 as capillary ECs^31^ (Fig. 2b, c). ENDO2 expressed a high level of *ACKR1* which was specially expressed in the human vein,^32^ suggesting ENDO2 as venous ECs (Fig. 2b, c). ENDO3, marked by *CCL21* and podoplanin (*PDPN)*, was identified as lymphatic ECs^14^ (Fig. 2b, c). Recent research has reported that RGCC and ACKR1 play roles in ECM organization.^8,33^ ECs primarily proliferated at 10 dpi and 12 wpi (Fig. 2f, Tables 3 and 4), with ENDO1 expanding at 10 dpi (Fig. 2e). After tendon injury, total three stages comparing with normal peritendinous tissue, the upregulated signaling pathways of ECs in tendon adhesion tissue including ECM–receptor interaction and focal adhesion (Fig. 2g).

**Fig. 3:**
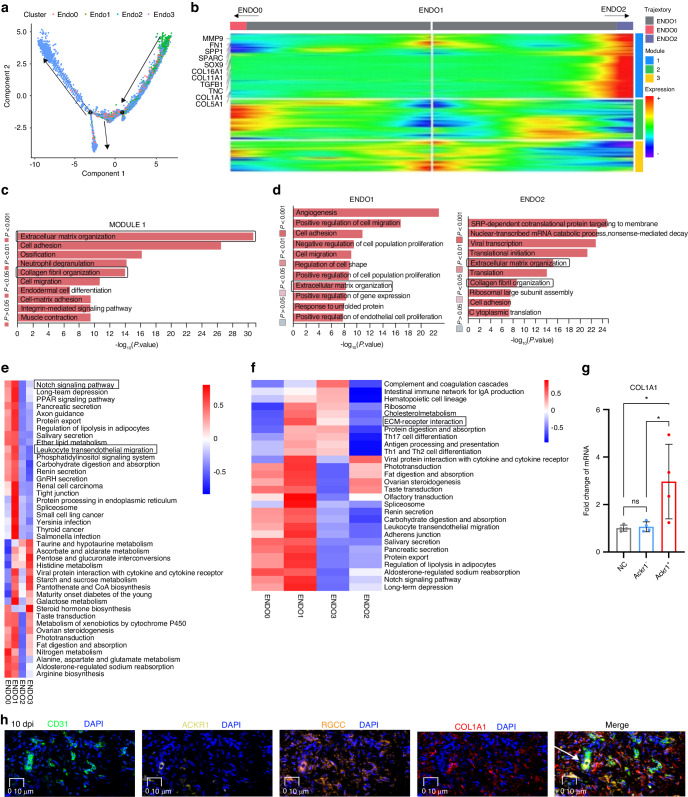
Identifying the profibrotic ECs. **a** The pseudotemporal trajectory analysis of ENDO0, ENDO1 and ENDO2. Arrows indicated the direction of pseudotemporal trajectory. **b** Heatmap of differential gene modules across ENDO1 to ENDO2 (right arrow) and ENDO1 to ENDO0 (left arrow) pseudotemporal trajectories. Grouped by hierarchical clustering (*n* = 3). The genes of module 1 were labeled at left. **c** The TOP 15 Gene Ontology enrichment of all genes in module 1, along ENDO0 to ENDO2 pseudotemporal trajectory. **d** The Gene Ontology enrichment analysis of ENDO1 (left) and ENDO2 (right). **e** The Qusage analysis of enriched pathways of each cluster of ECs. The color indicates mean pathway intensity (Red, high. Blue, low). Right, pathways. Bottom, clusters. **f** The KEGG analysis of enriched pathways of each cluster of ECs. The color indicates mean pathway intensity (Red, high. Blue, low). Bottom, clusters. Right, pathways. **g** Primary human fibroblasts treated with conditioned media from ENDO2 (ACKR1^+^ ECs) (*n* = 3) or ACKR1^-^ ECs (*n* = 3), qPCR of stated genes, expression relative to *COL1A1* mean expression of control primary human fibroblasts (*n* = 3), Mean ± SEM. **h** The polychromatic immunofluorescence for *CD31* (marker of ECs), *ACKR1*, *RGCC*, and *COL1A1* showed RGCC^+^ACKR1^+^ ECs (white arrows) existed around collagen at 10 dpi. *RGCC* and *ACKR1* had part colocalization. White arrows CD31^+^RGCC^+^ACKR1^+^ cells. *CD31*(green), *RGCC* (orange), *ACKR1* (yellow), *COL1A1* (red), *DAPI* (blue), scale bars 10 μm

Further analysis of pseudotemporal trajectory suggested that following injury, a differentiation trajectory from ENDO1 into ENDO0 into ENDO2 and the origin of ENDO1 were endothelial progenitor cells, we constituted 3 distinct gene expression modules (Fig. 3a and Fig. S5A, B). The heatmap of differential genes module across ENDO1 to ENDO2 pseudotemporal trajectory suggested fibrotic genes including *SPP1*, *FN1*, *COL1A1*, and *MMP9* upregulated in the process^8,34^ (Fig. 3b). GO enrichment analysis of Module 1 revealed that genes involved in ECM organization were upregulated during this process (Fig. 3c). To better understand ENDO1 and ENDO2 phenotypes, Gene Ontology enrichment analysis revealed angiogenesis and ECM organization in ENDO1 and ECM organization and collagen fibril organization in ENDO2 (Fig. 3d). The marker pathway analysis suggested the ECM-receptor interaction in both ENDO1 and ENDO2 (Fig. S5C). The Qusage analysis of enriched pathways of ECs indicated the fibrosis-associated pathway including *NOTCH* signaling pathway was upregulating in ENDO1^8,35^ (Fig. 3e). The KEEG analysis revealed the enriched pathway of ECM-receptor interaction in ENDO1 and ENDO2 (Fig. 3f). A heatmap of TFs shows ENDO1 enriched profibrotic TFs including Jun proto-oncogene (*JUN)*^36^ and ENDO2 enriched profibrotic TFs including Fos proto-oncogene (*FOS)*^37^ (Fig. S5D).

Our cellular experiments demonstrated that ACKR1^+^ endothelial cells promote fibrosis. We isolated and cultured ACKR1^+^ ECs from human 10 dpi tissue, and in vitro experiments showed that these cells increased the mRNA level of *COL1A1* in human fibroblasts (Fig. 3g and Fig. S9A). The presence of ACKR1^+^ ECs was adjacent to COL1A1, involving in fibrotic niche of human tendon adhesion tissue at 10 dpi (Fig. 3h). Additionally, the colocalization of CD31, RGCC, ACKR1, and GJA4 at 10 dpi suggested that GJA4^+^ ECs and RGCC^+^ ECs might transform into ACKR1^+^ ECs during tendon adhesion progression (Fig. 3h and Fig. S5E). Collectively, we identified ECs subsets in human tendon adhesion tissue, and our experiments indicated that ACKR1^+^ ECs contribute to fibrosis in human tendon adhesion.

### Distinct MP clusters are presented in tendon adhesion tissue

**Fig. 4:**
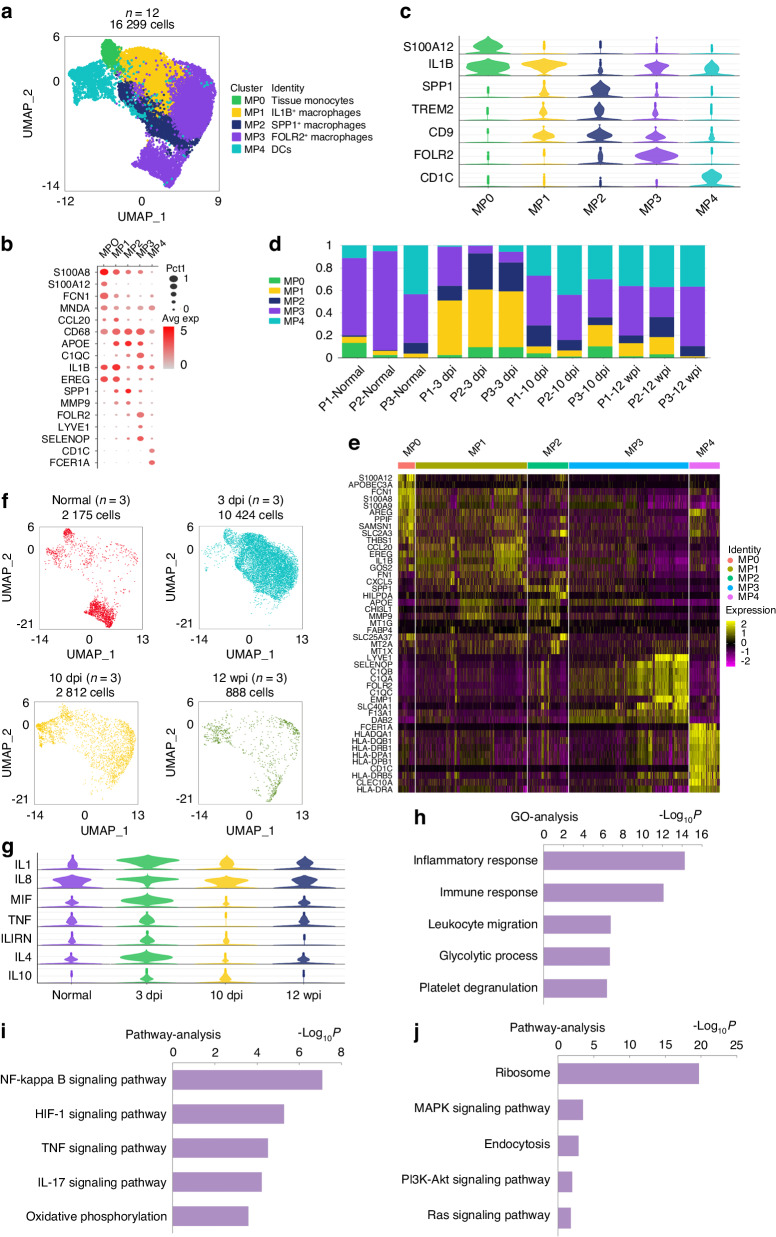
Distinct MP clusters present in human tendon adhesion tissue. **a** Clustering 16 299 mononuclear phagocytes (MP) from total 12 patients. DCs, dendritic cells. **b** Dot plot: showing cell clusters of MPs by known markers. The dot size indicates the gene expression percent in each cluster. The color indicates mean gene expression (Red, high). **c** The violin plot of selected gene expression of each cluster in MPs. **d** Bar plots of proportion of each cluster in MPs from 12 patients. **e** Heatmap of marker genes in each MP cluster. Top, clusters. Left, marker genes. **f** UMAP plots of MPs of each time point: normal, 3 dpi,10 dpi, and 12 wpi. **g** The violin plot of expression levels of pro- and anti-inflammatory cytokines in MPs, including pro-inflammatory cytokines such as IL1, IL8, MIF, TNF, and anti-inflammatory cytokines such as IL1RN, IL4, IL10. **h** The GO analysis of up-regulated genes of all three stages after tendon injury. **i** Pathway analysis of up-regulated signaling pathway of all three stages after tendon injury. **j** Pathway analysis of down-regulated signaling pathway of all three stages after tendon injury

**Fig. 5:**
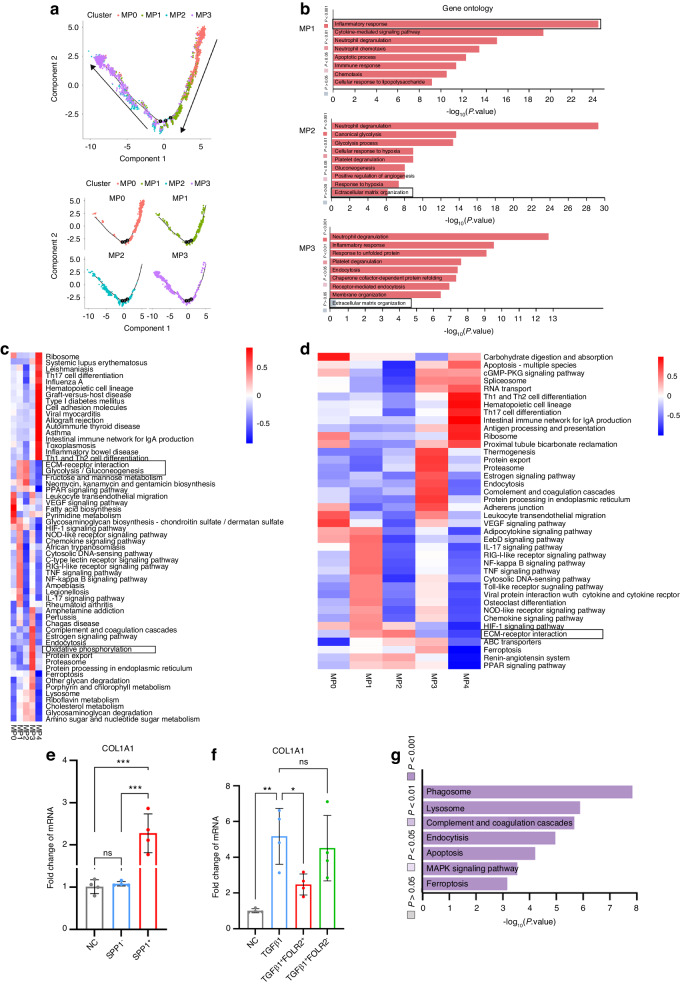
Identifying the profibrotic macrophages and antifibrotic macrophages. **a** The pseudotemporal trajectory analysis of MP0, MP1, MP2, and MP3. Arrows indicated the direction of pseudotemporal trajectory. **b** The Gene Ontology enrichment analysis of MP1, MP2 and MP3. **c** The Qusage analysis of enriched pathways of each cluster of MPs. The color indicates the mean pathway intensity (Red, high. Blue, low). Right, pathways. Bottom, clusters. **d** The KEGG analysis of enriched pathways of each cluster of MPs. The color indicates the mean pathway intensity (Red, high. Blue, low). Bottom, clusters. Right, pathways. **e** Primary human fibroblasts treated with conditioned media from MP2 (SPP1^+^macrphages) (*n* = 3) or SPP1^-^ macrophages (*n* = 3), qPCR of stated genes, expression relative to *COL1A1* mean expression of control primary human fibroblasts (*n* = 3), Mean ± SEM. **f** Primary human fibroblasts treated with TGFβ1 and conditioned media from MP3 (FOLR2^+^macrophages) (*n* = 3) or FOLR2^-^ macrophages (*n* = 3), qPCR of stated genes, expression relative to *COL1A1* mean expression of control primary human fibroblasts (*n* = 3), Mean ± SEM. **g** The pathway analysis of MP3

In our study, we characterized five MPs subsets that included dendritic cells (DCs), monocytes and macrophages (Fig. 4a). MP4 was defined as DCs marked CD1c molecule (*CD1C)* and Fc epsilon receptor Ia (*FCER1A)*^38^ (Fig. 4b, e). MP0 was defined as monocytes enriched in S100 calcium binding protein A8 (*S100A8)* and S100 calcium binding protein A12 (*S100A12)*^38–40^ (Fig. 4b, e). MP1, MP2 and MP3 were defined as macrophages marked *CD68*, apolipoprotein E (*APOE)* and complement C1q A chain (*C1QA)*^41^ (Fig. 4b). MP1 enriched by interleukin 1 beta *(IL1B)* and epiregulin (*EREG)* and the GO Ontology (GO) analysis revealed the MP1 participated in inflammatory response (Fig. 5b). Therefore, MP1 were defined as proinflammatory macrophages. MP2, characterized as profibrotic macrophages, was marked by secreted phosphoprotein 1 (*SPP1)* and matrix metallopeptidase 9 (*MMP9)*^8^ (Fig. 4b, c). MP3 was defined as antifibrotic macrophages expressing folate receptor beta (*FLOR2)* and lymphatic vessel endothelial hyaluronan receptor 1 (*LYVE1)* by our in vitro experiment^42,43^ (Fig. 4b, c). Normal peritendinous tissue primarily consisted of MP3, making up 70.9% of total MPs (Fig. 4d and Table 5). After the tendon injury, MP1 and MP2 increased significantly at 3 dpi, accounting for 48.9% and 15.9%, respectively, while FOLR2 proportion decreased to 30.2% (Fig. 4d and Table 5). Tendon injury led to a reduction in MP3 abundance within adhesion tissue, and this stage exhibited the highest macrophage count during adhesion progression (Fig. 4f). The MPs’ expression levels of pro- and anti-inflammatory cytokines at four distinct stages showed the cytokines had the highest levels at 3 dpi (Fig. 4g), indicating the inflammatory follow tendon injury was the most active at 3 dpi. As adhesion progressed, MP1 and MP2 proportions decreased, while MP3 increased at 10 dpi (Fig. 4d and Table 5). Macrophage numbers gradually declined, and by 12 weeks, granulation tissue formed with fewer macrophages. MP3 proportion among macrophages neared that of 10 dpi but remained lower than in normal peritendinous tissue (Fig. 4d and Table 5). After tendon injury, total three stages comparing with normal peritendinous tissue, the GO analysis of MPs revealed the inflammation relative response had upregulated (Fig. 4h), the upregulated signaling pathways of MPs in tendon adhesion tissue including HIF-1 signaling pathway, TNF signaling pathway and IL-17 signaling pathway (Fig. 4i), which were reported in tendon injury.^44^ By comparing with normal peritendinous tissue, the downregulated signaling pathways of MPs including MAPK signaling pathway after the tendon injury (Fig. 4j).

**Table 5:** MP clusters composition of total samples at different stages

Sample ID	MP0/%	MP1/%	MP2/%	MP3/%	MP4/%
Total normal	11.3	5.2	1.4	70.9	11.3
Total 3 dpi	3.7	48.9	15.9	30.2	1.3
Total 10 dpi	7.8	14.1	10.6	37.6	29.8
Total 12 wpi	1.5	9.5	10.6	41.3	36.1

### Profibrotic phenotype of SPP1^+^ macrophages

In a recent investigation, TREM2^+^SPP1^+^ macrophages in human fibrotic liver tissue were identified as scar-associated macrophages that could promote hepatic fibrosis.^8^ In the present study, we had identified MP2 marked *SPP1*, triggering receptor expressed on myeloid cells 2 *(TREM2)* and *MM9* in human tendon adhesion tissue (Fig. 4b, c). However, the ontogeny of human peritendinous macrophage subpopulations remains unclear. To further investigate MP2 origin, we visualized the pseudotemporal trajectory. These analyses indicated that upon injury, a differentiation trajectory progressed from MP0 to MP1, then MP1 to MP2 (Fig. 5a and Fig. S6A, B), the origin of MP0 was circulating monocytes in blood, suggesting that MP2 was monocyte-derived, consistent with previous research.

To further characterize the MP2 phenotype, we analyzed the heatmap of genes expressed by MP2 and observed upregulation of the profibrotic genes *SPP1*, *TREM2*, and CD9 molecule (*CD9)*^8,45^ (Fig. 4c). The results of GO analysis indicated the MP2 participated in extracellular matrix (ECM) organization (Fig. 5b), and the KEEG analysis suggested that the cells were involved in ECM–receptor interaction (Fig. 5d). The marker pathway analysis suggested the ECM-receptor interaction in MP2 (Fig. S6C). The Qusage analysis of pathways enriched in each MP subset showed that fibrosis-associated pathways including ECM–receptor interaction and glycolysis were upregulated in MP2 (Fig. 5c). Glycolysis was associated with inflammatory macrophages.^46^ The gene enrichment of MP2 ECM-receptor interaction hallmark gene set suggested fibrotic genes including *SPP1* and fibronectin 1 (*FN1)* upregulated (Fig. S6D). The gene enrichment of MP2 glycolysis hallmark gene set suggested glyceraldehyde-3-phosphate dehydrogenase (*GAPDH)*, lactate dehydrogenase A (*LDHA)* and enolase 1 (*ENO1)* upregulated (Fig. S6E). The heatmap of transcription factors (TFs) shows MP2 enriched fibrosis related TFs including ETS proto-oncogene 1 (*ETS1)*^47^ and glycolysis related TFs including basic helix-loop-helix family member e40 (*BHLHE40)*, SRY-box transcription factor 4 (*SOX4)*, SRY-box transcription factor 10 (*SOX10)*,^48–50^ MAPK signaling pathway related TFs including AP-1 transcription factor subunit (*JUN*) and MYC proto-oncogene (*MYC)* (Fig. S6G).

**Fig. 6:**
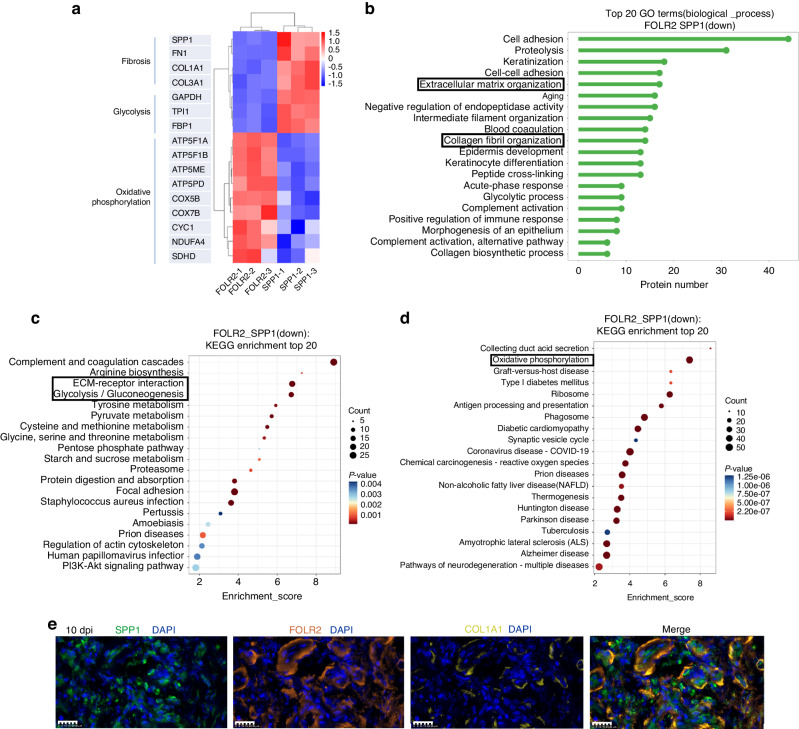
Proteomics and immunofluorescence of SPP1^+^ macrophages and FOLR2^+^ macrophages. **a** The heatmap of differentially expressed proteins between human FOLR2^+^ macrophages and SPP1^+^ macrophages. The color indicates the level of differential protein expression (Red, high. Blue, low). Left, the name of protein. Bottom, sample ID. **b** The TOP 20 Gene Ontology enrichment (biological process) of upregulated proteins in human SPP1^+^macrophages. **c** The KEGG analysis of Top20 enriched pathways of upregulated proteins in human SPP1^+^macrophages. The size of the bubble indicates the number of proteins (Big, many. Little, few). The color of bubble indicates the *P* value of pathway (Red, low. Blue, high). Bottom, enrichment score. Left, pathways. **d** The KEGG analysis of Top20 enriched pathways of upregulated proteins in human FOLR2^+^macrophages. The size of bubble indicates the number of proteins (Big, many. Little, few). The color of bubble indicates the *P* value of pathway (Red, low. Blue, high). Bottom, enrichment score. Left, pathways. **e** Representative polychromatic immunofluorescence images of 10 dpi human tendon adhesion tissue: *SPP1* (green), *FOLR2* (orange), *COL1A1* (the marker to collagen, yellow), *DAPI* (blue), scale bars 25 μm. Immunofluorescence showed *SPP1* and *FOLR2* expression was around collagen at 10 dpi stage

Our analyses revealed that MP2’s involvement in ECM-receptor interaction suggested a potential profibrotic function. Subsequently, we isolated and cultured SPP1^+^ macrophages from human 3 dpi tissue, and the in vitro experiments showed that SPP1^+^ macrophages upregulated the mRNA level of collagen type I alpha 1 chain (*COL1A1)* in human fibroblasts (Fig. 5e and Fig. S9A). To compare the cross-species, we performed scRNA-seq on mouse tendon adhesion tissue. We collected the tendon adhesion tissue from the mouse model of tendon adhesion by injuring the flexor digitorum longus tendon. Finally, we clustered and annotated 19 919 mouse MPs from mouse tendon adhesion tissue including five distinct stages: normal peritendinous tissue, 3, 7, 14 and 28 days post injury. Five MPs clusters were identified including MP2 marked by *SPP1*, MP3 marked by *FOLR2* and selenoprotein P (*SELENOP)* (Fig. S7A–C). The Qusage analysis of enriched pathways in mouse MPs suggested that MP2 was involved in ECM–receptor interaction and glycolysis (Fig. S7D). The results suggested the mouse MP2 was similar to human MP2, SPP1^+^ macrophages in tendon adhesion were conserved across species. We also conducted a comparative proteomic analysis between SPP1^+^ and FOLR2^+^ macrophages from human 10 dpi tissue, obtaining a total of 1 851 proteins, including 515 upregulated proteins and 444 downregulated proteins in FOLR2^+^ macrophages (Fig. S6H). The heatmap of differentially expressed proteins indicated upregulation of profibrotic proteins including *SPP1*, *FN1*, *COL1A1*, and *COL3A1*, along with glycolysis proteins including *GAPDH*, triosephosphate isomerase 1 (*TPI1)* and fructose-bisphosphatase 1 (*FBP1)* in SPP1^+^ macrophages (Fig. 6a). The biological process GO terms of differentially expressed proteins suggested that ECM organization and collagen fibril organization were upregulated in SPP1^+^ macrophages (Fig. 6b). The KEGG enrichment of differentially expressed proteins showed the ECM-receptor interaction and glycolysis upregulated in SPP1^+^ macrophages (Fig. 6c). Immunofluorescence analysis demonstrated the presence of human SPP1^+^ macrophages surrounding collagen at 10 dpi (Fig. 6e and Fig. S8C). At other stages revealed that SPP1^+^ macrophages were sparse in normal peritendinous tissue and proliferated at the 3 dpi (Fig. S8A–D).

In conclusion, our study showed that SPP1^+^ macrophages in tendon adhesion tissue originated from monocytes and exhibited a profibrotic phenotype, expanding early during the human tendon adhesion process, were conserved across species.

### Antifibrotic phenotype of FOLR2^+^ macrophages

Our research identified MP3 as antifibrotic macrophages in tendon adhesion, marked by *FOLR2*, *LYVE1* and *SELENOP* (Fig. S7B, C). In normal peritendinous tissue, MP3 represented the largest proportion of macrophages, which decreased substantially at 3 dpi due to early inflammation after tendon injury (Fig. 4d).

GO Ontology enrichment analysis indicated MP3’s involvement in neutrophil degranulation (Fig. 5b). Qusage analysis of enriched pathways across different MPs subsets revealed upregulation of oxidative phosphorylation in MP3, which is associated with anti-inflammatory macrophages,^46^ while fibrosis-related pathways were minimally present in MP3 (Fig. 5c). Previous research has suggested that oxidative phosphorylation could reduce hepatic fibrosis.^51^ The gene enrichment of MP3 oxidative phosphorylation hallmark gene set suggested the relative genes including cytochrome c oxidase subunit 5B (*COX5B)*, cytochrome c oxidase subunit 7B (*COX7B)* and ATP synthase membrane subunit e (*ATP5ME)* upregulated (Fig. S6F). KEEG analysis revealed the enriched pathway of endocytosis and thermogenesis in MP3 (Fig. 5d). The pathway analysis of MP3 revealed MAPK signaling pathway upregulated (Fig. 5g). A heatmap of TFs shows MP3 enriched TFs including MYC proto-oncogene (*MYC)* which related with oxidative phosphorylation and MAPK signaling pathway^52^ (Fig. S5d). Intriguingly, our cell experiments confirmed that FOLR2^+^ macrophages exhibited an antifibrotic phenotype. We isolated and cultured FOLR2^+^ macrophages from human 10 dpi tissue, and in vitro experiments demonstrated that FOLR2^+^ macrophages reduced mRNA level of *COL1A1* in human fibroblasts (Fig. 5f and Fig. S9A). Then we identified mouse MP3 marked by *FOLR2* and *SELENOP* (Fig. S7A-C). The heatmap of metabolism pathways in mouse MPs suggested that MP3 was involved in oxidative phosphorylation (Fig. S7E). The results suggested the mouse MP3 was similar to human MP3, FOLR2^+^ macrophages in tendon adhesion were conserved across species. Proteomics of SPP1^+^ and FOLR2^+^ macrophages supported our findings, as the heatmap of differentially expressed proteins suggested that oxidative phosphorylation-related proteins including ATP synthase F1 subunit alpha (*ATP5F1A)*, ATP synthase F1 subunit beta (*ATP5F1B)*, *ATP5ME*, ATP synthase peripheral stalk subunit d *(ATP5PD)*, *COX5B*, *COX7B*, cytochrome c1 (*CYC1)*, NDUFA4 mitochondrial complex associated (*NDUFA4)* and succinate dehydrogenase complex subunit D (*SDHD)* were upregulated in FOLR2^+^ macrophages^51^ (Fig. 6a). The KEGG enrichment of differentially expressed proteins suggested oxidative phosphorylation upregulated in FOLR2^+^ macrophages (Fig. 6d), suggesting the anti-fibrotic property of FOLR2^+^ macrophages might through its highly active oxidative phosphorylation feature. Furthermore, MP3 accounted for a lot in normal peritendinous tissue, the MAPK signaling pathway was downregulated in all three stages after tendon injury and upregulated in MP3, these may support MP3 antifibrotic through the MAPK signaling pathway.

Immunofluorescence revealed the presence of human FOLR2^+^ macrophages surrounding collagen at 10 dpi (Fig. 6e and Fig. S8C), and throughout various stages of tendon adhesion, including normal, 3 dpi, 10 dpi, and 12 wpi (Fig. S8A–D). In conclusion, our study showed that FOLR2^+^ macrophages in tendon adhesion tissue originate from monocytes and exhibit an antifibrotic phenotype during the tendon adhesion process and repair after tendon injury during three stages, which were conserved across species. Oxidative phosphorylation or MAPK signaling pathway is a potential pathway that could explain this antifibrotic phenotype. Our findings suggest that FOLR2^+^ macrophages help prevent excessive fibrosis in the human tendon adhesion process.

### ACKR1 participated in FOLR2^+^ macrophages migration

**Fig. 7:**
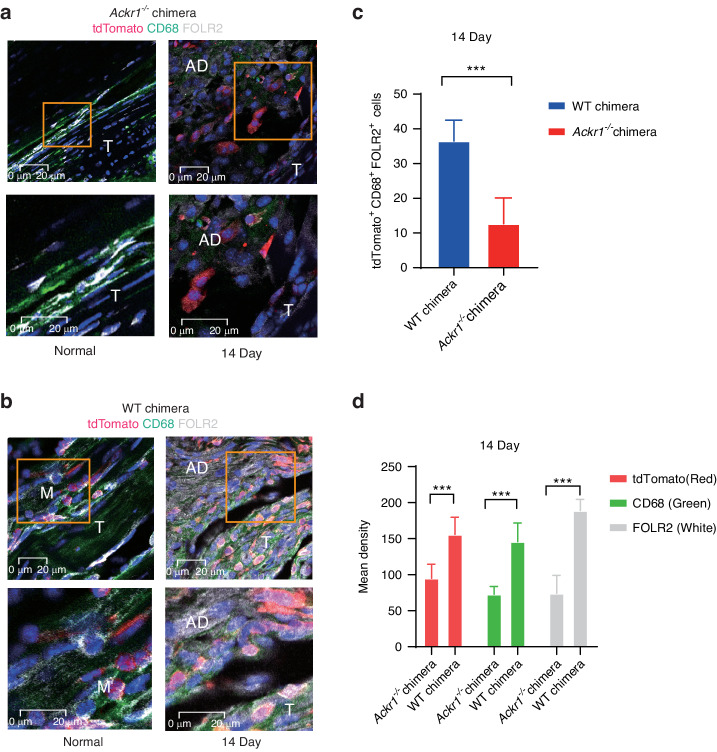
The immunofluorescence images of *Ackr1*^*−/−*^ and WT chimeras**. a** The polychromatic immunofluorescence for *tdTomato* (the marker to monocytes), *CD68* and *FOLR2* of *Ackr1*^*−/−*^ chimeras. *Tdtomato* (red), *CD68* (green), *FOLR2* (white), *DAPI* (blue), scale bars 20 μm. T, tendon. M, muscle. AD, adhesion tissue. **b** The polychromatic immunofluorescence for *tdTomato*, *CD68* and *FOLR2* of *Ackr1*^*−/−*^ chimeras. *tdTomato* (red), *CD68* (green), *FOLR2* (white), *DAPI* (blue), scale bars 20 μm. T tendon. M muscle. AD adhesion tissue. **c** The number of tdTomato^+^CD68^+^FOLR2^+^ cells of WT chimeras (*n* = 6), *Ackr1*^*−/−*^ chimeras (*n* = 6). Mean ± SEM. **d** The mean density of immunofluorescence for *tdTomato*, *CD68*, and *FOLR2* of WT chimeras (*n* = 6) and *Ackr1*^*−/−*^ chimeras (*n* = 6)

The recent study revealed ACKR1 could support monocyte migration.^9^ Based on the study, we generated chimeras mice by bone-marrow transfer from *Lysm-Cre;R26R*^*tdTomato*^ mice to lethally irradiated *Ackr1*^*−/−*^ mice (*Ackr1*^*−/−*^ chimeras; deficient in ACKR1) and control mice (WT chimeras). Immunofluorescence analysis revealed CD68^+^FOLR2^+^ monocytes decreased in *Ackr1*^*−/−*^ chimeras in tendon adhesion of 14 days post injury (Fig. 7a–d). The result demonstrated ACKR1 participated in FOLR2^+^ macrophages migration in tendon adhesion.

### Deciphering the intricate interplay among multiple cell lineages in the peritendinous fibrotic niche

**Fig. 8:**
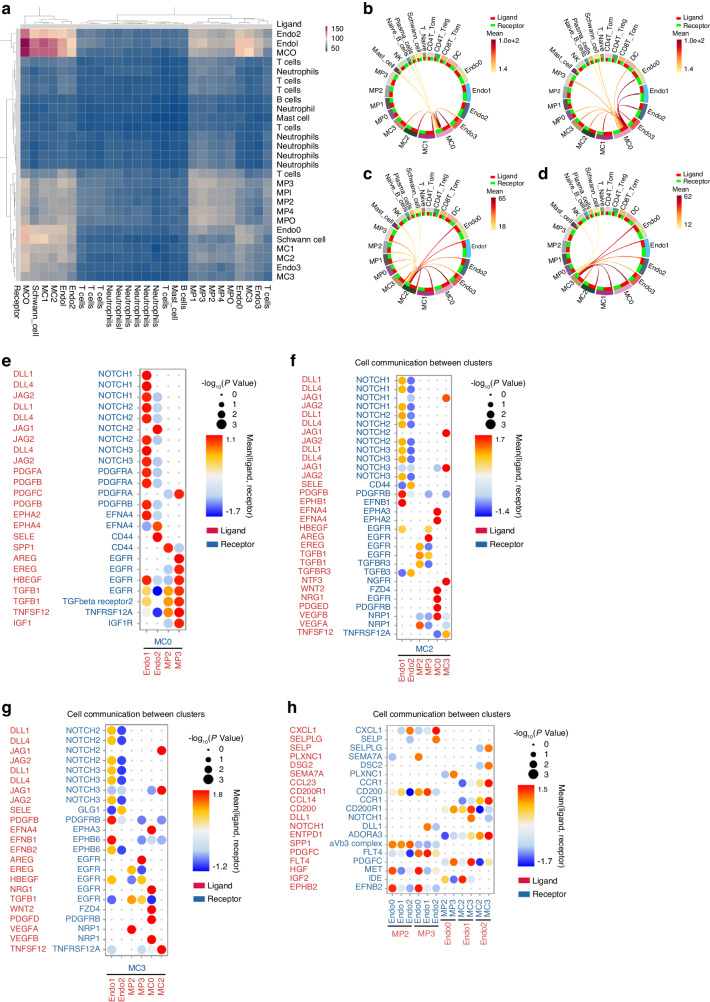
Characterization of the cellular interactome in the human tendon adhesion. **a** Heatmap of interactions between all clusters. Left, ligands. Bottom, receptors. **b** Circosplot of the interactions between MC0 and other clusters at 10 dpi. **c** Circosplot of the interactions between MC2 and other clusters at 10 dpi (Red, high. Yellow, low). **d** Circosplot of the interactions between MC3 and other clusters at 10 dpi (Red, high. Yellow, low). **e** Dotplot of ligand-receptor interactions between MC0 and ENDO1, ENDO2, MP2, MP3. Size of the circle indicated *P* value (Big, high. Small, low). **f** Dotplot of ligand-receptor interactions between MC2 and ENDO1, ENDO2, MP2, MP3, MC3. Size of circle indicated *P* value (Big, high. Small, low). **g** Dotplot of ligand-receptor interactions between MC3 and ENDO1, ENDO2, MP2, MP3, MC3. Size of circle indicated *P* value (Big, high. Small, low). **h** Dotplot of ligand-receptor interactions between MP and ENDO. Size of circle indicated *P* value (Big, high. Small, low)

To further explore multi-lineage interactions in human tendon adhesion tissue, we employed cellphoneDB to reveal interactions among various fibrotic clusters, and found frequent cell interactions between ENDO1, ENDO2 and MCs in human tendon adhesion tissue (Fig. 8a). The circle plot also revealed strong cellular interactions between ENDO1, ENDO2 and MC0 at each stage of tendon adhesion (Figs. 8b and Fig. S9B–E). MPs, ECs and MCs interacted with MC2 at 10 dpi stage (Fig. 8c). Meanwhile, ECs and MCs interacted with MC3 at 10 dpi stage (Fig. 8d). In detail, ENDO1 expressed high level of ligands delta like canonical Notch ligand 1 (*DLL1)*, delta like canonical Notch ligand 4 (*DLL4)*, jagged canonical Notch ligand 1 (*JAG1)* and jagged canonical Notch ligand 2 (*JAG2)* which were interacted with *NOTCH* receptors including notch receptor 1 (*NOTCH1)*, notch receptor 2 (*NOTCH2)* and notch receptor 3 (*NOTCH3)* on MC0 (Fig. S8E). In addition, ENDO1 expressed high level of ligand *PDGFB* and interacted with receptors platelet-derived growth factor receptor alpha (*PDGFRA)* and platelet-derived growth factor receptor beta (*PDGFRB)* on MC0 (Fig. 8e). ENDO2 expressed a high level of selectin E (*SELE)*, the recent study revealed them as adhesion molecules which mediate adhesion and rolling of leukocytes on ECs^53^ (Fig. 8e). Both MP2 and MP3 expressed high level of ligands *TGFB1* (Fig. 8e). MP2 might contribute to fibrosis in human tendon adhesion through the ligands *SPP1, EREG,* and *TGFB1* (Fig. 8e). MP3 expressed high level of ligand *IGF1* to receptor *IGFR* on MC0 and *IGF1-IGFR* was reported in MAPK signaling pathway (Fig. 8e). The recent study reported *IGF1* from macrophages could promote muscle regeneration and *IGF1* could heal tendon lesion.^54–56^ MP3 may inhibit fibrosis by *IGF1* through MAPK signaling pathway to promote tendon healing. Furthermore, the cellular interactions between MC2, MC3 and ENDO1, ENDO2, MC0 showed similar results (Fig. 8f, g). Interestingly, ENDO1 expressed the ligand *PDGFB*, MP2 expressed ligands *TGFB1* and vascular endothelial growth factor A (*VEGFA)*, MC0 expressed ligands platelet-derived growth factor D (*PDGFD)* and vascular endothelial growth factor B (*VEGFB)* with respective receptors on MC2 and MC3 (Fig. 8f, g). *TGFB*,^57,58^ *PDGF,* and *VEGF*^59^ are all known to be involved in PMT. Our findings support the notion that PMT in human tendon adhesion might be driven by RGCC^+^ ECs, SPP1^+^ macrophages and ADAM12^+^ fibroblasts through their respective pathways. Furthermore, MP3 specifically expressed ligand C-X-C motif chemokine ligand 1 (*CXCL1)* on receptor ACKR1 in ENDO2 (Fig. 8h), that indicated ACKR1 may be involved in the migration of FOLR2^+^ macrophages by ligand CXCL1.

In summary, our cellphoneDB analysis of multiple key clusters within human tendon adhesion tissue revealed potential pathways that contribute to fibrosis. Our results revealed that MPs, ECs and MCs communicate in the fibrotic niche to facilitate fibrogenic progression. Among this process, *NOTCH* and *PDGF* signaling might be pivotal during ECs to MCs communication, while *EREG* and *TGFB1* signaling might be important for MPs to MCs to facilitate tendon adhesion. MP3 interacted with MC0 by *IGF1*. Furthermore, we demonstrated that ACKR1 may be involved in the migration of FOLR2^+^ macrophages by ligand CXCL1.

## Discussion

Tendon adhesion limits the daily movement greatly and finally leads to severe disability.^2^ The mechanism underlying tendon adhesion was still not fully understood. Here, multi-omics analysis including scRNA-seq and proteomics was performed on both human and mouse tendon adhesion tissue to identify the single-cell transcriptomic atlas at different stages after human tendon injury. The transcriptomes of over 74 000 human single cells were profiled. We found that SPP1^+^ macrophages, RGCC^+^ endothelial cells, ACKR1^+^ endothelial cells and ADAM12^+^ fibroblasts promote fibrosis of tendon tissue and that the cellular composition varied depending on all three stages after tendon injury. Notably, the FOLR2^+^ macrophages had an antifibrotic function. Furthermore, ACKR1 was verified to regulate FOLR2^+^ macrophages migration in the injured peritendinous site through generating chimeras mice by bone-marrow transfer from *Lysm-Cre;R26R*^*tdTomato*^ mice to lethally irradiated *Ackr1*^*−/−*^ mice (*Ackr1*^*−/−*^ chimeras; deficient in ACKR1) and control mice (WT chimeras). FOLR2 and *Tdtomato* can be simultaneously observed in specific cells.

Macrophages were widely known as a trigger of adhesion formation, although exact clusters and function are still unclear.^10^ Among them, recent studies revealed SPP1^+^ macrophages promoted hepatic fibrosis and pulmonary fibrosis.^8,45^ SPP1^+^ macrophages were also identified as profibrotic cluster by in vitro experiments. These macrophages were verified to induce fibrosis through interaction with fibroblasts via *EREG* and *TGFB1*. Interestingly, FOLR2^+^ macrophages were identified as antifibrotic cluster. *FOLR2*, folate receptor beta, encodes for the folate family, which exhibits a high affinity for folic acid and several reduced folic acid derivatives.^42^ Recent studies have reported several functions of FOLR2^+^ macrophages in other diseases. For example, a positive association has been reported between FOLR2^+^ macrophages and cancer prognosis.^43,60^ In our study, by combining mouse tendon adhesion scRNA-seq, proteomics, immunofluorescence images, and in vitro cell experiments, we proposed that FOLR2^+^ macrophages could mitigate fibrosis through direct interaction with fibroblasts via pathways such as oxidative phosphorylation or MAPK signaling pathway and *IGF1* was the possible ligand. Therefore, FOLR2^+^ cluster and FOLR2 target which could be a potential avenue for tendon adhesion treatment.

Endothelial cells were detected in multiple organs fibrosis and adhesion tissues, although their function is unknown. For example, *RGCC* was linked to renal fibrosis, while ACKR1^+^ ECs promoted pulmonary fibrosis.^61,62^ Besides, the similar profibrotic phenomenon of ACKR1^+^ ECs was also found in hepatic fibrosis.^8^ In our recent study, ACKR1^+^ ECs were identified as profibrotic clusters and further certification was performed. We demonstrated RGCC^+^ ECs may be involved in fibrosis via *PDGFB* and *NOTCH* signaling pathways while ACKR1^+^ ECs promoted fibrosis through interaction with fibroblasts via *SELE*. ACKR1 was reported to participate in neutrophil migration.^9^ Notably, we revealed ACKR1 involved in FOLR2^+^ macrophages migration. By generating *Ackr1*^*−/−*^ chimeras, we observed the significant decline of FOLR2^+^ macrophages, indicating that ACKR1 was specifically involved in FOLR2^+^ macrophage migration. CXCL1-ACKR1 was the possible ligand-receptor between FOLR2^+^ macrophages and ACKR1^+^ ECs.

Other types of cells and associated clusters were also found. ADAM12^+^ fibroblasts were previously considered as progenitors of a major proportion of collagen-overproducing cells during the process of scarring and synovial fibrosis.^24,63^ In this study, they were uncovered as multipotent stromal cells in tendon adhesion. Intriguingly, we integrated this finding from cellphoneDB with immunofluorescence images and discovered that PMT might occur in human tendon adhesion, regulated by SPP1^+^ macrophages, RGCC^+^ ECs, and ADAM12^+^ multipotent stromal cells. However, the intensive study of ADAM12^+^ multipotent stromal cells in tendon adhesion was insufficient. Thus, ADAM12^+^ multipotent stromal cells should be investigated in further study. Our study still had some flaws, such as the limited number of samples. In future studies, additional time points after tendon injury should also be considered.

In conclusion, our research presents the multi-omics study including a single-cell transcriptomic atlas and describes the fibrosis microenvironment landscape of human tendon adhesion based on scRNA-seq of human tendon adhesion tissue at four distinct stages. ACKR1^+^ ECs and FOLR2^+^ macrophages were respectively identified as antifibrotic and profibrotic phenotypes. Interestingly, ACKR1^+^ ECs participated in FOLR2^+^ macrophages migration in tendon adhesion. Overall, our study uncovers a clear cluster and novel mechanism of human tendon adhesion mainly by identifying FOLR2^+^ macrophages, migrated by ACKR1, as peritendinous antifibrotic macrophages. They could serve as potential therapeutic targets for human tendon adhesion treatment.

## Materials and methods

### Study design

The objective of this study was to investigate the fibrosis microenvironment of tendon adhesion at three stages after tendon injury. We collected 12 samples at four distinct stages of tendon adhesion to perform scRNA-seq and compare respective characteristics at the cellular level. The sample size was based on previous studies and the difficult collections of human tendon adhesion tissue.

### Study subjects

#### Human subjects

Human tendon adhesion and normal peritendinous tissue was surgically resected from flexor tendon tenolysis patients at Wuxi Ninth People’s Hospital (Wuxi, China). All patients were recruited from orthopedics of Wuxi Ninth People’s Hospital. The Ethical Committee of Wuxi Ninth People’s Hospital approved the study and all patients signed informed consent. The number is KT201803. Table 1 summarized the pathological and clinical information from a total of 12 patients. The criterion for tissue collection is as follow. The peritendinous granulation adhesion tissue was scarped around tendon trauma without tendon tissue.

#### Mouse subjects

All the mice experiments accorded with the guidelines published by NIH and Shanghai Sixth People’s Hospital Internal Review Board (Shanghai, China). All C57BL/6J mice were purchased from the animal facility of Shanghai Sixth People’s Hospital. Constructing the mouse tendon adhesion model, narcotizing the 10-week-old male mice by 3% entobarbital sodium. Then disinfected the skin in the right hind-paw by 75% ethanol. Sectioning the skin and exposing the flexor digitorum longus tendon. Then cut off the tendon and repaired it by modified Kessler pattern, using 8-0 sutures. Finally, close the wound with 6-0 sutures. All the mice were treated as equal and allowed to move freely in the cage.

#### Single-cell separation of human tendon adhesion tissue

The primary tendon adhesion tissue and normal peritendinous tissue were resected from flexor tendon tenolysis patients and tendon adhesion mice. Then washed with PBS and removed the muscle tissue and fat tissue. The rest tissue was cut into small pieces. Then shook tissue in 15 mL of EDTA-containing buffer (5 mmol/L EDTA, 15 mmol/L HEPES, 1 mmol/L DTT and 10% FBS-supplemented PBS) for 1 h at 37 °C. Then the tissue was digested by collagenase I (Worthington, LS004196) at 3 mg/mL, neutral protease (Solarbio, D6430) at 4 mg/mL and DNase I (Solarbio, D8071) at 30 U/mL for 75 min at 37 °C until the tissue was digested completely. After digestion, filtered cells through a 100 μm filter and washed with PBS. Then discarded the supernatant and resuspended the precipitates with red blood cell lysis buffer for 5 min. We then washed the cells with HBSS containing 1% FBS and 2 mmol/L EDTA. Finally, filtered cells through a 30 μm filter. Dissociated single cells were then stained with AO/PI for viability assessment using Countstar Fluorescence Cell Analyzer.

#### Single-cell RNA sequencing

The scRNA-Seq libraries were generated using the 10X Genomics Chromium Controller Instrument and Chromium Single Cell 3′ V3.1 Reagent Kits (10X Genomics, Pleasanton, CA). Concentrated cells to ~1 000 cells/μL. Then loaded the cells into each channel to generate single-cell Gel Bead-In-Emulsions (GEMs). The RT step broke GEMs and the barcoded-cDNA was purified and amplified. The amplified barcoded cDNA was fragmented, A-tailed, ligated with adapters and index PCR amplified. Quantified the final libraries by using the Qubit High Sensitivity DNA assay (Thermo Fisher Scientific) and determined the size distribution of the libraries by using a High Sensitivity DNA chip on a Bioanalyzer 2200 (Agilent). Sequenced all libraries by illumina sequencer (Illumina, San Diego, CA) on a 150 bp paired-end run.

#### Single-cell RNA sequencing data processing and analysis

scRNA-seq data analysis was performed by NovelBio Co., Ltd. with NovelBrain Cloud Analysis Platform (www.novelbrain.com). We applied fastp with default parameter to filter the adapter sequence and the low quality reads were removed to achieve clean data. Then we used CellRanger v5.0.1 to obtain the feature-barcode matrices by aligning reads to the human genome (GRCh38 Ensemble: version 100).

To achieve the aggregated matrix, we applied the down sample analysis among samples sequenced according to the mapped barcoded reads per cell of each sample. Cells contained over 200 expressed genes and controlled the mitochondria UMI rate below 20% by passing the cell quality filtering. Then removed mitochondria genes in the expression table. Used Seurat package (version: 4.0.3, https://satijalab.org/seurat/) for cell normalization and regression were based on the expression table according to the UMI counts of each sample and percent of mitochondria rate to obtain the scaled data. Constructed PCA which was based on the scaled data with top 2 000 high variable genes and used top 10 principals for UMAP construction. The unsupervised cell cluster result was acquired based on the PCA top 10 principals by utilizing graph-based cluster method. Then the marker genes were calculated by FindAllMarkers function with wilcox rank sum test algorithm under the following criteria:1. lnFC > 0.25; 2. *P* value < 0.05; 3. min.pct > 0.1. We selected the clusters of the same cell type for re-UMAP analysis, graph-based clustering and marker analysis to identify the cell type in detail.

#### Pseudo-time analysis and RNA velocity

We applied the Single-Cell Trajectories analysis by utilizing Monocle2 (http://cole-trapnell-lab.github.io/monocle-release) using DDR-Tree and the default parameter. We selected marker genes of the Seurat clustering result and raw expression counts of the cell passed filtering before Monocle analysis. We applied branch expression analysis modeling (BEAM Analysis) for branch fate determined gene analysis based on the pseudo-time analysis. We annotated the spliced reads and un spliced reads using the velocyto python package based on previous aligned bam files of SCRNA-seg data to perform the RNA velocity analysis. The calculation of RNA velocity values for each gene in each cleaned embedding RNA velocity vector to low-dimension space were done by following the scvelo python pipeline. The velocity-based cell transition matrix was calculated by using the transition matrix function from scvelo. We estimated the destination of a cell by identifying the highest correlation value in the matrix. Then a Fisher’s exact test was performed on 2 × 2 cluster-by-cluster or cluster-by-tissue contingency tables to test fate destinations of cell clusters of interest.

#### Cell communication analysis

We applied cell communication analysis based on the CellPhoneDB, a public repository of ligands, receptors and their interactions to analyze the cell-cell communication molecules systematically. We annotated membrane, secreted and peripheral proteins of the cluster of different time point. Based on the interaction and the normalized cell matrix achieved by Seurat Normalization, we calculated Significant mean and Cell Communication significance (*P* value < 0.05).

#### SCENIC analysis

The Single-cell regulatory network inference and clustering (pySCENIC, v0.9.5) (Aibar et al., 2017) workflow was applied and used the 20-thousand motifs database for RcisTarget and GRNboost to assess transcription factor regulation strength.

#### QuSAGE analysis (gene enrichment analysis)

QuSAGE (2.16.1) analysis was performed to characterize the relative activation of a given gene set.

#### Co-regulated gene analysis

We used find_gene_modules function of monocle3 with the default parameters to discover the gene co-regulation network.

#### Go analysis

We performed Gene ontology (GO) analysis to facilitate elucidating the biological implications of marker genes and differentially expressed genes. The GO annotations were downloaded from NCBI (http://www.ncbi.nlm.nih.gov/), UniProt (http://www.uniprot.org/) and the Gene Ontology (http://www.geneontology.org/). We applied the Fisher’s exact test to identify the significant GO categories and FDR was used to correct the *P* value.

#### Pathway analysis

We used pathway analysis to find out the significant pathway of the marker genes and differentially expressed genes according to KEGG database. Then we turn to the Fisher’s exact test to select the significant pathway, and defined the threshold of significance by *P* value and FDR.

#### Proteomics

Sorted SPP1^+^ macrophages and FOLR2^+^macrophages from human tendon adhesion tissue at 10 dpi, respectively. Then preprocessed the samples and used timsTOF Pro2 spectrometry platform to obtain raw data by DIA mode. Each sample was repeated for triple. Then matched raw data with directDIA spectra, extracted quantitative information and performed statistical analysis. Annotate functions on identified proteins by several databases and performed GO analysis and Pathway analysis on the differential proteins.

#### Polychromatic immunohistochemistry

The polychromatic immunohistochemistry was performed by a Five-color Fluorescence kit (Recordbio Biological Technology, Shanghai, China) based on the tyramide signal amplification technology according to the manufacturer’s instruction. Briefly, 4-μm paraffin tissue sections were dewaxed with xylene for 15 min and then cleared in 100% ethanol for 15 min. Then performed antigen retrieval with PH 6.0 sodium citrate solution. The sections were blocked by endogenous peroxidase with 3% hydrogen peroxide and washed in PBS for 15 min. Then the sections were blocked with 3% BSA-PBS for 30 min at room temperature and incubated with primary antibody for 2 h at 37 °C. Then washed sections with PBS for 15 min and incubated with secondary antibody for 50 min at room temperature. The sections were washed with PBS for 15 min and developed with TYR fluorescent dye for 15 min. Then washed sections with PBS for 15 min and repeated steps from the antigen retrieval step until all antigens were stained. Finally, washed sections with PBS for 15 min and counterstained with DAPI for 10 min at room temperature. Mounted the sections with anti-fluorescence quenching regent. Tissue sections were imaged using a fluorescence microscope. Used a 20× objective to capture fluorescence images and collected the image data by using NIS Elements (Nicon, V4.50.00). Used Imaris 9.0.1 for the analysis of image data.

The following antibodies were used: SPP1, ThermoFisher, MA5-29580; FOLR2, BioLegend, 391703; MNDA, Novus, NBP2-57799; COL1A1, Novus, NB600-408; CD31, BioLengd, 303103 RGCC, Bioss, bs-9079R; COL4A1, NOVUS, NBP1-97716; ACKR1, NOVUS, NB100-2421;GJA4, ThermoFisher, 42-4400; CXCL14, Abcam, ab264467; ADAM12, Abcam, ab39155; COL4A1, Novus, NBP1-97716;MYH11, Abcam, ab133567.

#### Flow cytometry and cell sort

Briefly, washed fresh cells from 10 dpi human tendon adhesion tissue with PBS for 10 min firstly. Then filtered cell through a 100 μm filter with 5 mL PBS. Washed cells with PBS for 5 min and centrifugated. The supernatant was discarded and resuspended the precipitate with 500 μL PBS. Then cells were incubated with primary antibody for 30 min at 4 °C. Then add 1.5 mL PBS and centrifugated. Discarded the supernatant and resuspended the precipitate with 500 μL PBS. The incubated cells with secondary antibody for 30 min at 4 °C. Then add 1.5 mL PBS and centrifugated. Discarded the supernatant and resuspended the precipitate with 1 mL PBS. Finally, filtered cells through a 70 μm filter and sorted the cells.

The following antibodies were used: CD68, BioLgend, 333807; SPP1, ThermoFisher, MA5-29580; FOLR2, BioLegend, 391703; CD31, BioLengd, 303103; ACKR1, NOVUS, NB100-2421 S100A4, BioLgend, 370005; ADAM12, Abcam, ab39155; Goat Anti-Rabbit IgG H&L (Alexa Fluor® 594), Abcam, ab150080; Rabbit Anti-Mouse IgG H&L (Alexa Fluor® 594), Abcam, ab150128.

### Cell co-culture

#### Human tendon adhesion tissue macrophage and fibroblast cell co-culture

Seeded SPP1^+^CD68^+^ macrophages (20 000 cells) and SPP1^-^CD68^+^ macrophages (20 000 cells) from human tendon adhesion tissue at 10 dpi into culture dishes with HFF1 human fibroblasts respectively. Co-cultured cells with cells in DMEM medium contain with 10% FBS and 1% Penicillin-Streptomycin for 72 h.

Seeded FOLR2^+^CD68^+^ macrophages (20 000 cells) and FOLR2^-^CD68^+^ macrophages (20 000 cells) from human tendon adhesion tissue at 10 dpi into culture dishes with HFF1 human fibroblasts respectively. After 24 h, added TGFβ1 (PEPROTECHA, 100-21) with 3 ng/mL. Co-cultured cells in DMEM medium contain with 10% FBS and 1% Penicillin–Streptomycin for 72 h.

#### Human tendon adhesion tissue endothelial cell and fibroblast cell co-culture

Seeded ACKR1^+^PECAM1^+^ endothelial cells (20 000 cells) and ACKR1^-^PECAM1^+^ endothelial cells (20 000 cells) from human tendon adhesion tissue at 10 dpi into culture dishes with HFF1 human fibroblasts respectively. Co-cultured cells in DMEM medium contain with 10% FBS and 1% Penicillin–Streptomycin for 72 h.

#### RNA extraction and RT-qPCR

Isolated RNA from human co-cultured cells by using RNAprep Pure Micro Kit (DP420, TIANGEN BIOTECH (BEIJING) CO., LTD) and performed cDNA synthesis by using HiScript III RT SuperMix for QPCR (Vazyme, R323-01). All steps were according to the manufacturer’s protocol. Then performed reactions in triplicate in 384-well plate format. Then performed RT-qPCR for human co-cultured cells by using ChamQ Universal SYBR qPCR Master Mix (Vazyme, Q711-02) with the following primers (all Sangon Biotech): *ACTB* (Forward CTTCGCGGGCGACGAT, Reversed ACATAGGAATCCTTCTGACCCAT), *COL1A1* (Forward GATTCCCTGGACCTAAAGGTGC, Reversed AGCCTCTCCATCTTTGCCAGCA).

Amplified the samples on an ABI 7900HT FAST PCR system (Applied Biosystems, ThermoFisher Scientific) and analyzed the data with ThermoFisher Connect cloud qPCR analysis software (ThermoFisher Scientific). Then used *ACTB* for normalization to quantify the 2^−ΔΔCt^. Estimated the amount of target mRNA in samples and expression calculated relative to average mRNA expression by using 2^−ΔΔCt^ quantification method.

#### Cell trilineage differentiation

Performed human ADAM12^+^ fibroblasts from 10 dpi human tendon adhesion tissue into adipogenic differentiation by using adipogenic differentiation kits (Oricell, HUXMX1-90031). Performed ADAM12^+^ fibroblasts into osteogenic differentiation by using osteogenic differentiation kits (Oricell, HUXMX1-900321). Performed ADAM12^+^ fibroblasts into chondrogenic differentiation by using chondrogenic differentiation kits (Oricell, HUXMX1-90041). All steps were according to the manufacturer’s protocol. Collected the image data by using NIS Elements (Nicon, V4.50.00). Used Imaris 9.0.1 for the analysis of image data.

#### Generation of bone marrow chimeric mice

Transfer bone marrow cells from *ACKR1*^*−/−*^ or WT mice into *Lysm-Cre;R26R*^*tdTomato*^ mice to generate mice which exhibit ACKR1-deficiency in the hematopoietic compartment and WT control chimeras. Irradiate the mice with two doses of 5 Gy and 4 h apart each time. Inject 1.5 × 10^6^ bone marrow cells from donor mice into irradiated mice the next day. After 4 weeks, performing IVM analysis of the chimeras.

### Statistical analysis

ImageJ, GraphPad Prism 8.0 and SPSS software (version 10.0, IBM Corp, Armonk, New York, USA) were used for statistical analysis. The results were presented as the mean ± SEM. Paired 2-tailed t tests were used for comparisons between two groups. A significant difference was considered when *P* < 0.05.

### Supplementary information

Supplemental Figures

## Data Availability

All data associated with this study are present in the paper or the Supplementary Materials. The scRNA-seq, this study is available at SRA: PRJNA975881, PRJNA976191.
