# Supplementary material for: Multi-omics analysis of human tendon adhesion reveals that ACKR1-regulated macrophage migration is involved in regeneration
Source: Bone Res. 2024 May 7;12:27. doi: 10.1038/s41413-024-00324-w (PMC11076548; doi:10.1038/s41413-024-00324-w)
Supplement: Supplementary file 1 — Supplemental Figures [file 41413_2024_324_MOESM1_ESM.docx]

**Supplemental Figures**

**
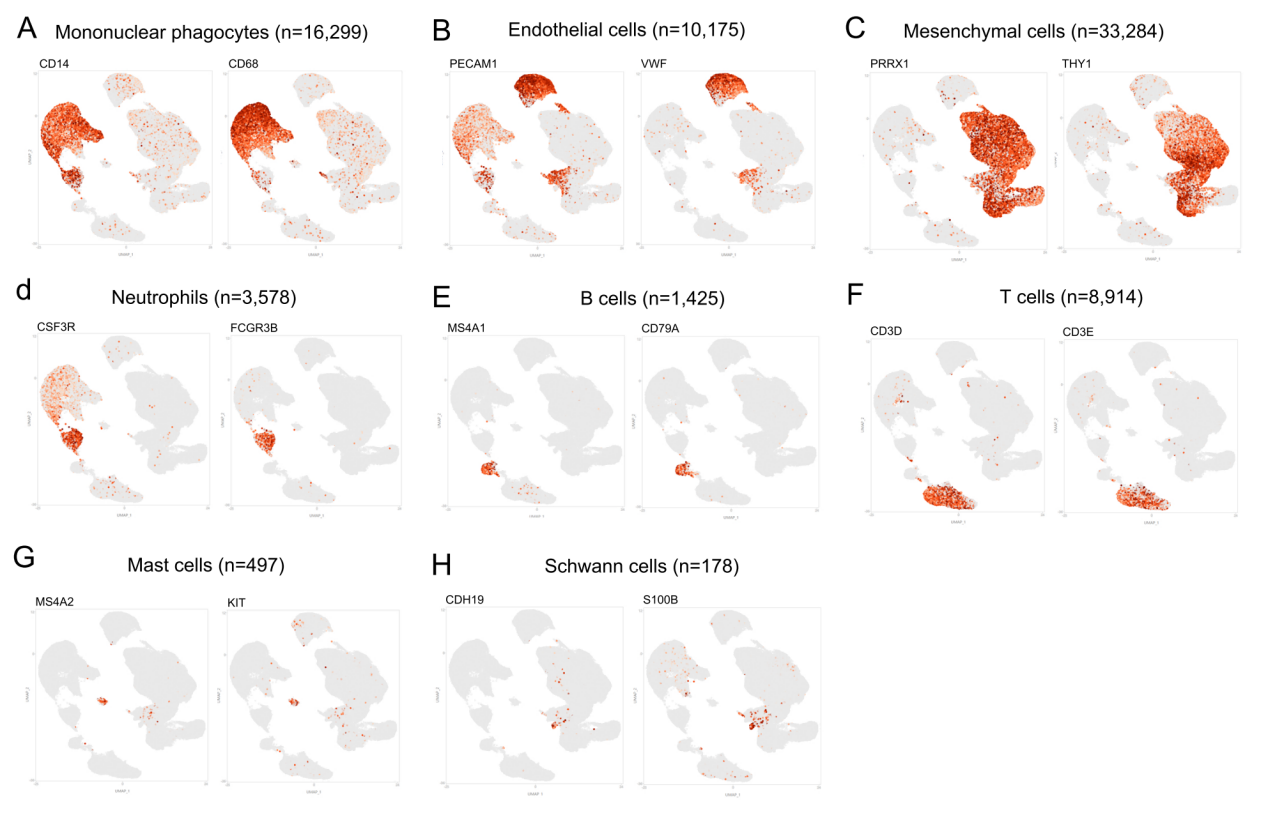
**

**Supplementary Figure 1. Expression of cell-type markers in total 8 clusters.**

(**A** to **H**) Marker gene expression of mononuclear phagocytes (**A**), endothelial cells (**B**), mesenchymal cells (**C**), neutrophils (**D**), B cells (**E**), T cells (**F**), mast cells (**G**) and schwann cells (**H**) on UMAP plots.


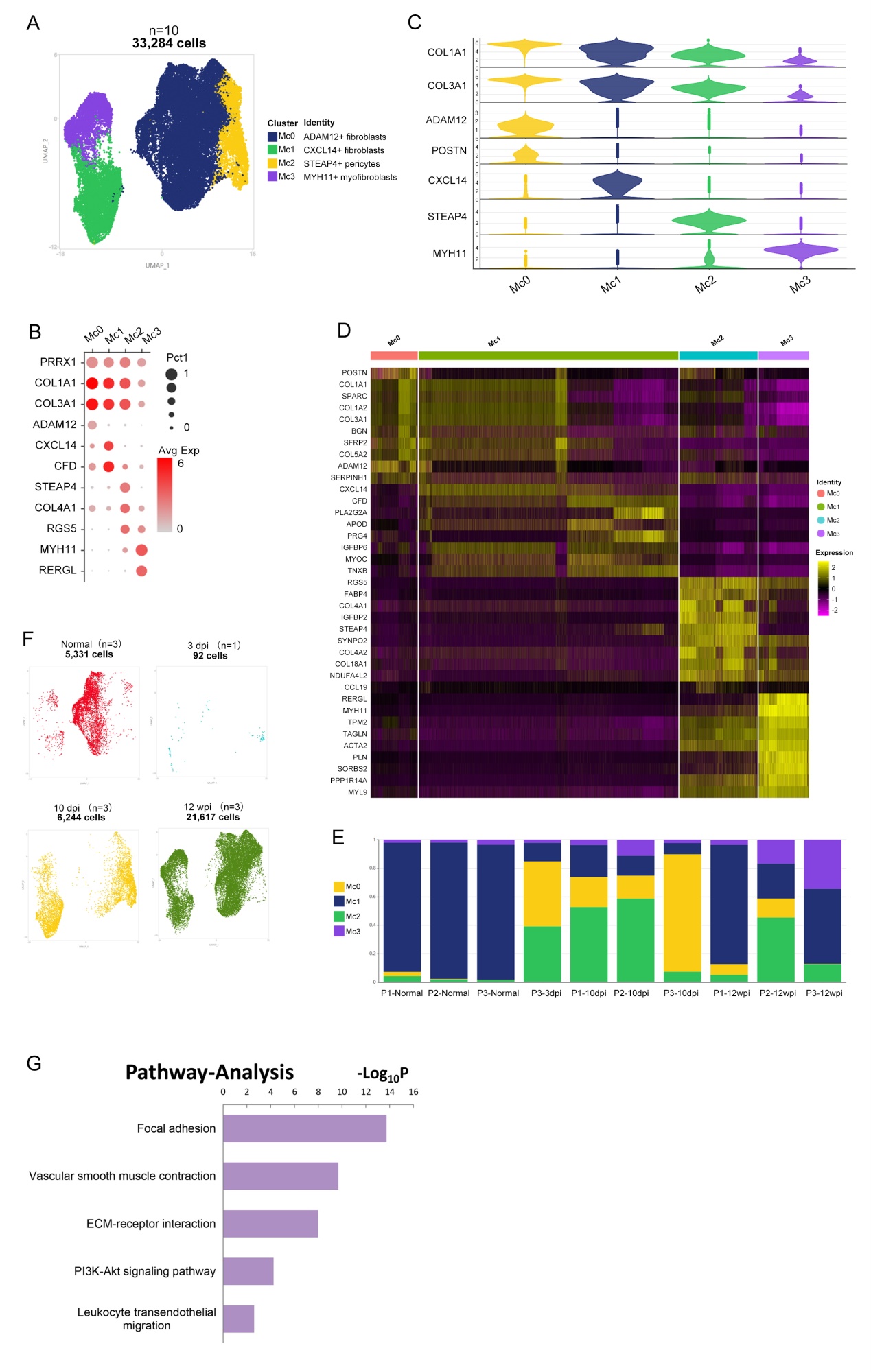


**Supplementary Figure 2. Distinct MC clusters present in human tendon adhesion tissue.**

(**A**) Clustering 33,284 mesenchymal cells (MCs) from total 10 patients. MC, mesenchymal cell.

(**B**) Dot plot: showing cell clusters of mesenchymal cells by known markers. The dot size indicates the gene expression percent in each cluster. The color indicates mean gene expression (Red, high).

(**C**) The violin plot of selected genes expression of each cluster in MCs.

(**D**) Heatmap of marker genes in each MC cluster. Top, clusters. Left, marker genes.

(**E**) Bar plots of proportion of each cluster in MCs from 10 patients.

(**F**) UMAP plots of MCs of each time point: normal, 3 dpi ,10 dpi and 12 wpi.

(**G**) Pathway analysis of up-regulated signaling pathway of all three stages after tendon injury.


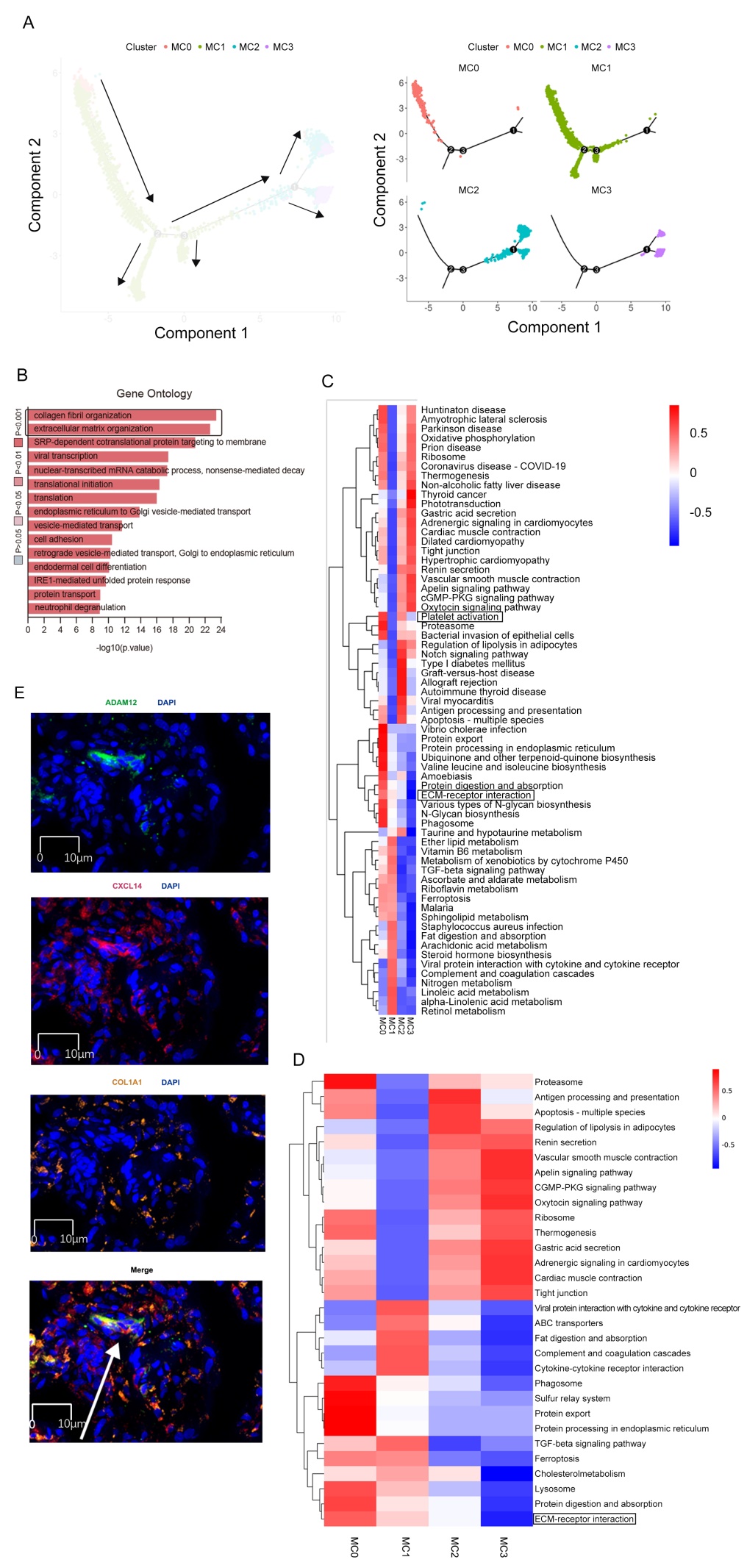


**Supplementary Figure 3. Identifying the profibrotic MCs.**

(**A**) The pseudotemporal trajectory analysis of MC0, MC1, MC2 and MC3. Arrows indicated the direction of pseudotemporal trajectory.

(**B**) The Gene Ontology enrichment analysis of MC0.

(**C**) The Qusage analysis of enriched pathways of each cluster of MCs. The color indicates mean pathway intensity (Red, high. Blue, low). Right, pathways. Bottom, clusters.

(**D**) The KEGG analysis of enriched pathways of each cluster of MCs. The color indicates mean pathway intensity (Red, high. Blue, low). Bottom, clusters. Right, pathways.

(**E**) The polychromatic immunofluorescence for *ADAM12*, *CXCL14* and *COL1A1* showed AMAM12^+^CXCL14^+^ cells (white arrows) existed around collagen at 10 dpi. *RGCC* and *ACKR1* had part colocalization. White arrows AMAM12^+^CXCL14^+^ cells. *ADAM12* (green), *CXCL14* (red), *COL1A1* (organ), *DAPI* (blue), scale bars 10μm.


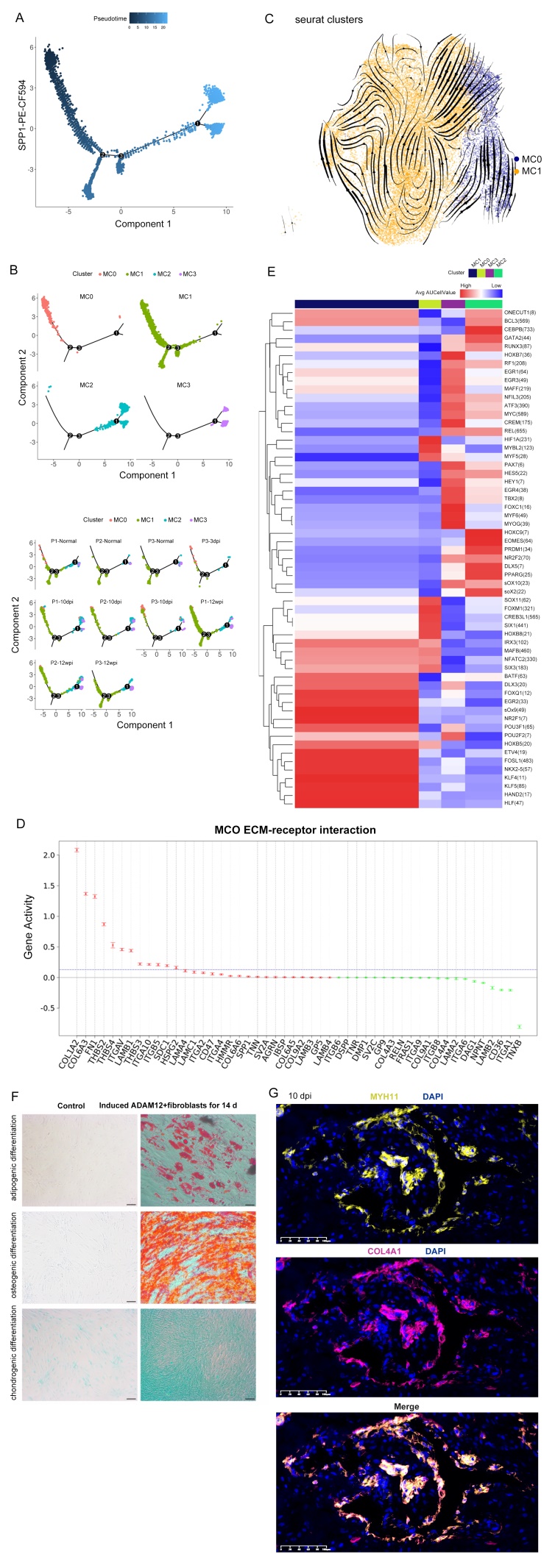


**Supplementary Figure 4. Annotating MCs.**

(**A**). The developmental pseudo-time of MC0, MC1, MC2 and MC3. The color indicated the progression (Dark, early. Bright, late).

(**B**) The pseudotemporal trajectory of MC0, MC1, MC2 and MC3 in each sample.

(**C**) The RNA velocity visualization of MC0 and MC1. Arrows indicated the progression.

(**D**) The gene activity in the ECM-receptor interaction gene set of MC0. Bottom, genes. Left, gene activity.

(**E**) Heatmap of TFs in each cluster of MCs. The color indicates mean TFs intensity (Red, high. Blue, low). Top, clusters. Right, TFs.

(**F**) Induced ADAM12^+^ fibroblasts into adipogenic, osteogenic and chondrogenic differentiation for 14 days. Left, control. Right, induced. Scale bars 100μm.

(**G**) The polychromatic immunofluorescence for *MYH11* (the marker to myofibroblasts) and *COL4A1* (the marker to pericytes) showed MYH11^+^COL4A1^+^cells existed at 10 dpi stage. *MYH11* and *COL4A1* had colocalization. *MYH11* (yellow), *COL4A1* (purple), *DAPI* (blue), scale bars 100μm.


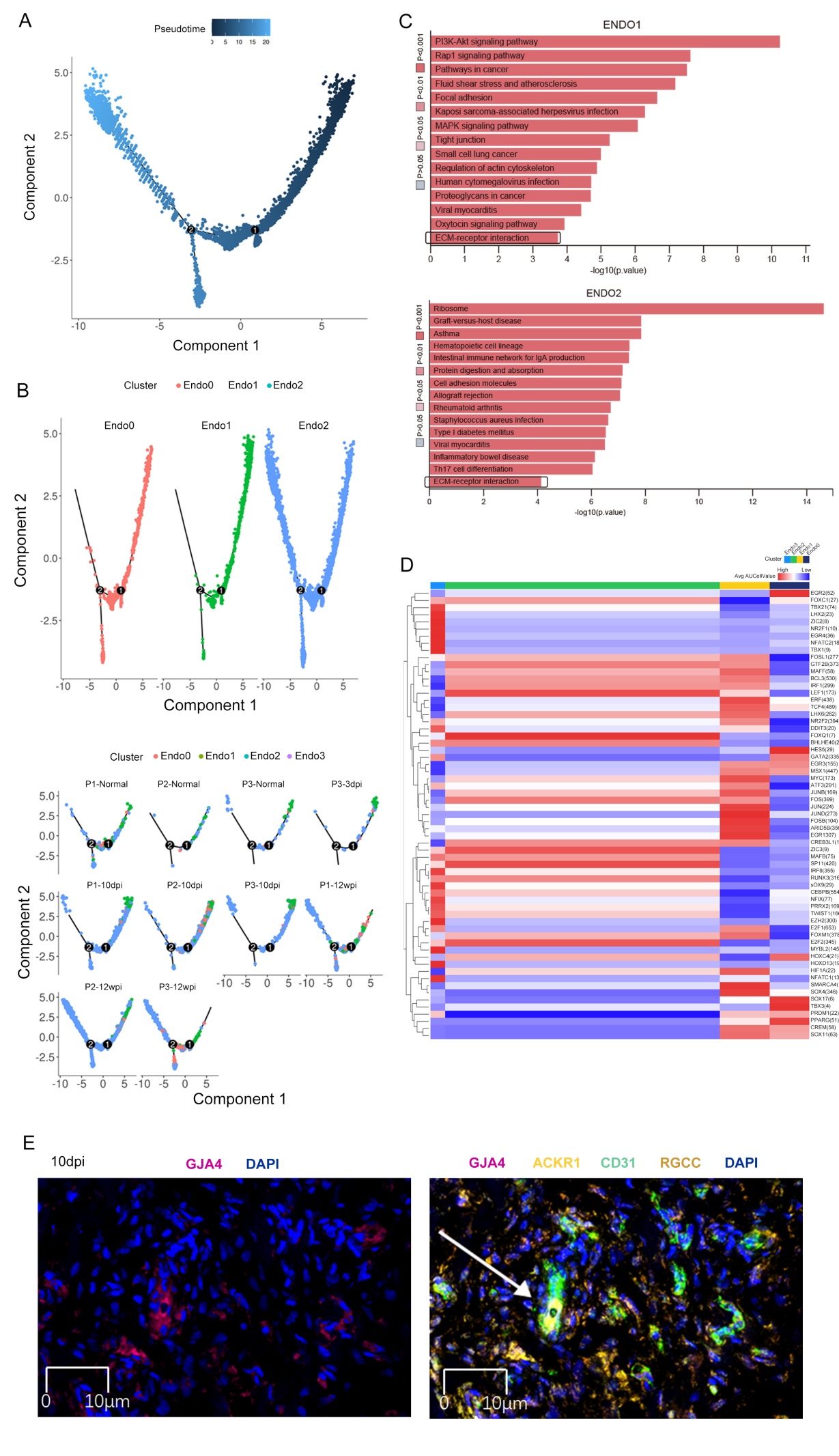


**Supplementary Figure 5. Annotating ENDO1 and ENDO2.**

(**A**) The developmental pseudo-time of ENDO0, ENDO1 and ENDO2. The color indicated the progression (Dark, early. Bright, late).

(**B**) The pseudotemporal trajectory of ENDO0, ENDO1 and ENDO2 in each sample.

(**C**) The TOP15 marker pathway analysis of ENDO1 (left) and ENDO2 (right).

(**D**) Heatmap of TFs in each cluster of ECs. The color indicates mean TFs intensity (Red, high. Blue, low). Top, clusters. Right, TFs.

(**E**) The polychromatic immunofluorescence for *GJA4*, *ACKR1*, *RGCC* and *CD31* (the marker to endothelial cells) showed GJA4^+^ACKR1^+^RGCC^+^CD31^+^cells existed at 10 dpi stage. *GJA4*, *ACKR1*, *RGCC* and *CD31* had colocalization. *GJA4* (purple), *ACKR1* (yellow), *RGCC* (orange), *CD31*(green), *DAPI* (blue), scale bars 10μm.


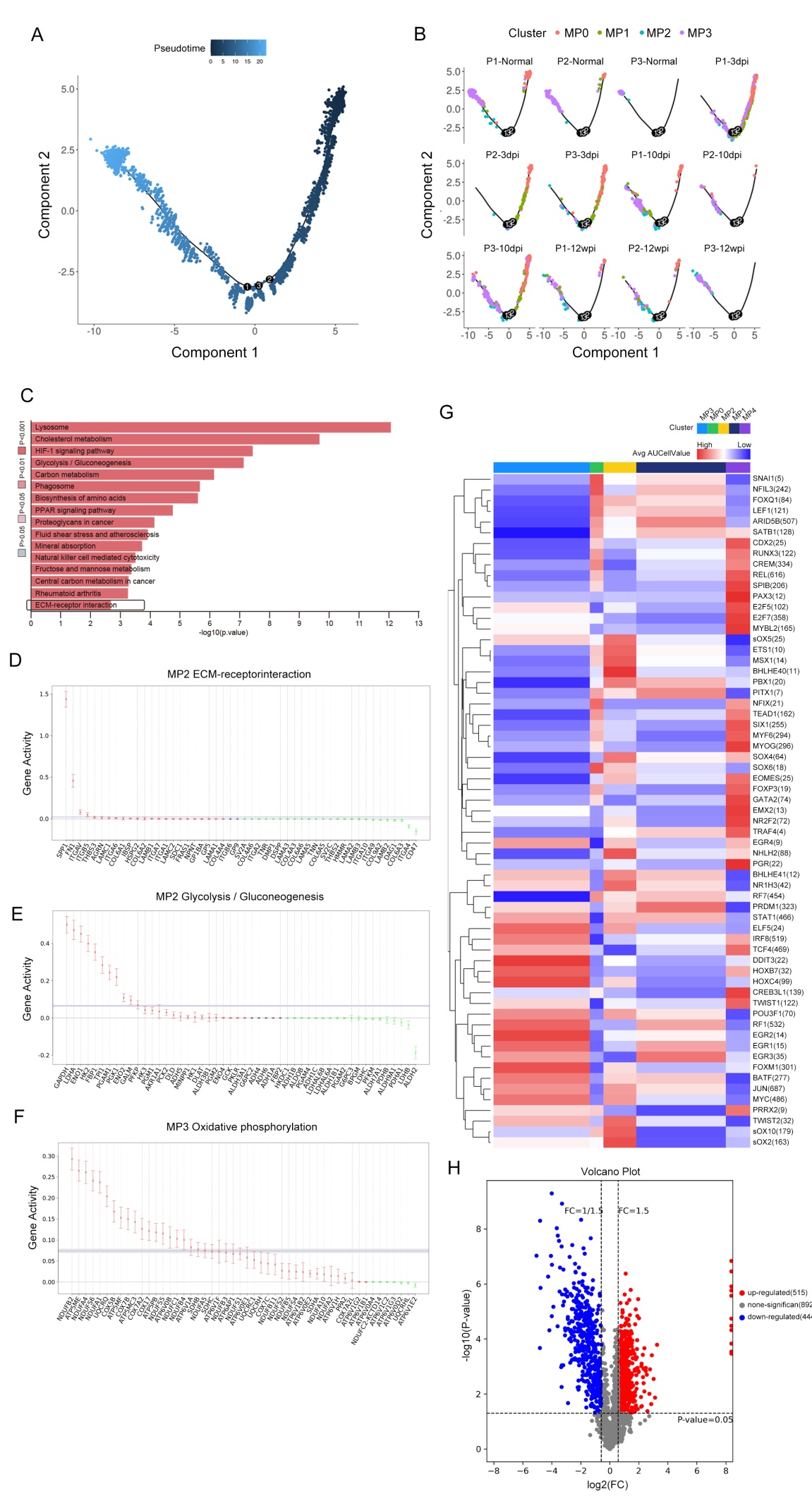


**Supplementary Figure 6. Annotating MP2 and MP3.**

(**A**) The developmental pseudo-time of MP0, MP1, MP2 and MP3.

The color indicated the progression (Dark, early. Bright, late).

(**B**) The pseudotemporal trajectory of MP0, MP1, MP2 and MP3 in each sample.

(**C**) The marker pathway analysis of MP2.

(**D**) The gene activity in the ECM-receptor interaction gene set of MP2. Bottom, genes. Left, gene activity.

(**E**) The gene activity in the glycolysis gene set of MP2. Bottom, genes. Left, gene activity.

(**F**) The gene activity in the oxidative phosphorylation gene set of MP3. Bottom, genes. Left, gene activity.

(**G**) Heatmap of transcription factors (TFs) in each cluster of MPs. The color indicates mean TFs intensity (Red, high. Blue, low). Top, clusters. Right, TFs.

(**H**) Volcano Plot of total 1,851 proteins in human FOLR2^+^macrophages and SPP1^+^macrophages. Red, up-regulated proteins in FOLR2^+^macrophages. Blue, down-regulated proteins in FOLR2^+^macrophages. Grey, none-significant proteins.


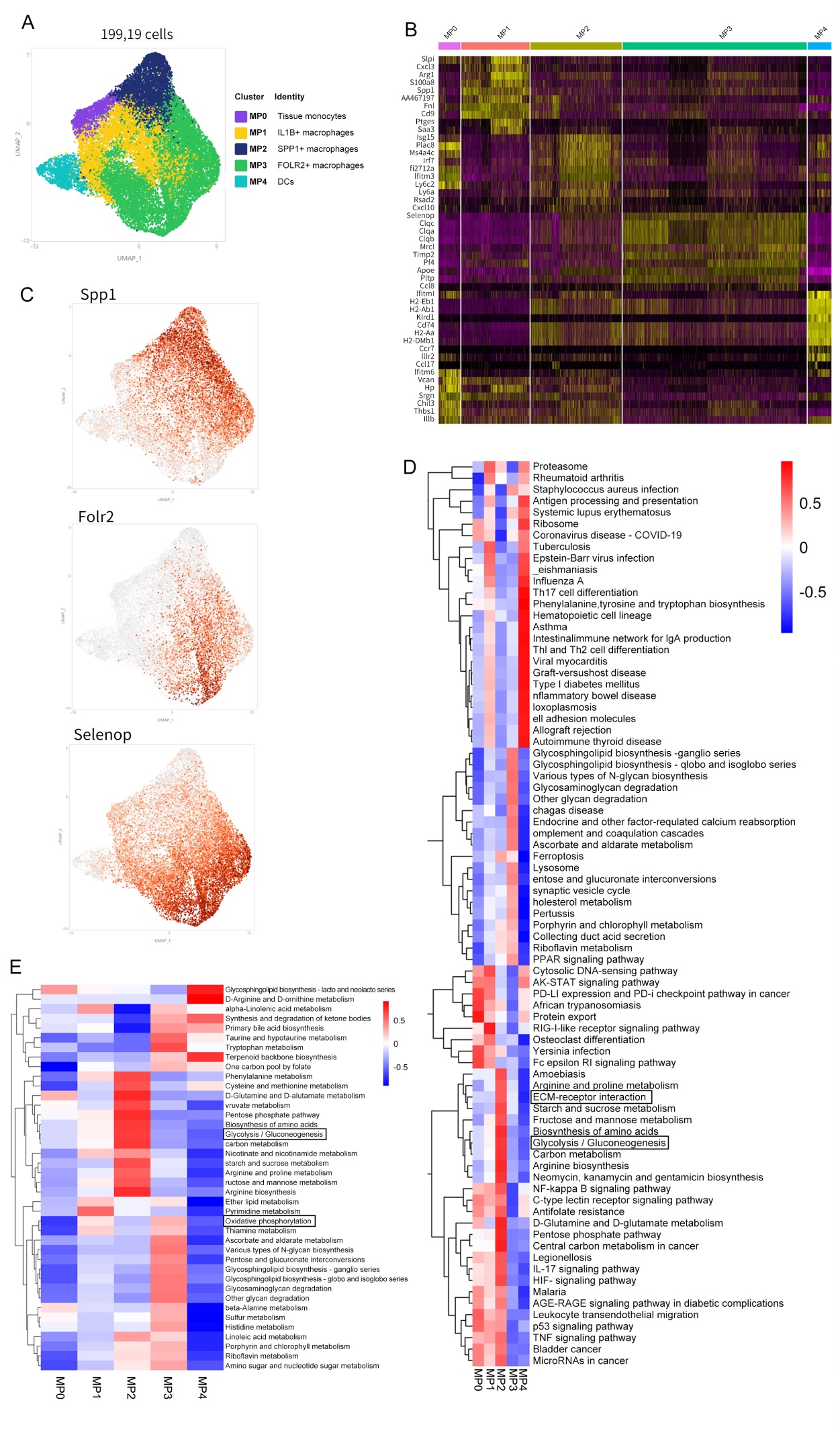


**Supplementary Figure 7. Characterisation of mononuclear phagocytes in mouse tendon adhesion.**

(**A**) Clustering and annotating 199,19 mouse mononuclear phagocytes (MPs) from mouse tendon adhesion tissue including five distinct stages: normal peritendinous tissue (n=1), 3 dpi (n=1), 7 dpi (n=1), 14 dpi (n=1) and 28 dpi (n=1). DCs, dendritic cells.

(**B**) Heatmap of marker genes in each mouse MP cluster.

Top, clusters. Left, marker genes.

(**C**) Marker gene expression of mouse MP2 and MP3 on UMAP plots.

(**D**) The Qusage analysis of enriched pathways of each cluster of mouse MPs. The color indicates mean pathway intensity (Red, high. Blue, low). Right, pathways. Bottom, clusters.

(**E**) The heatmap of metabolism pathways of each cluster in mouse MPs. The color indicates mean pathway intensity (Red, high. Blue, low). Right, pathways. Bottom, clusters.


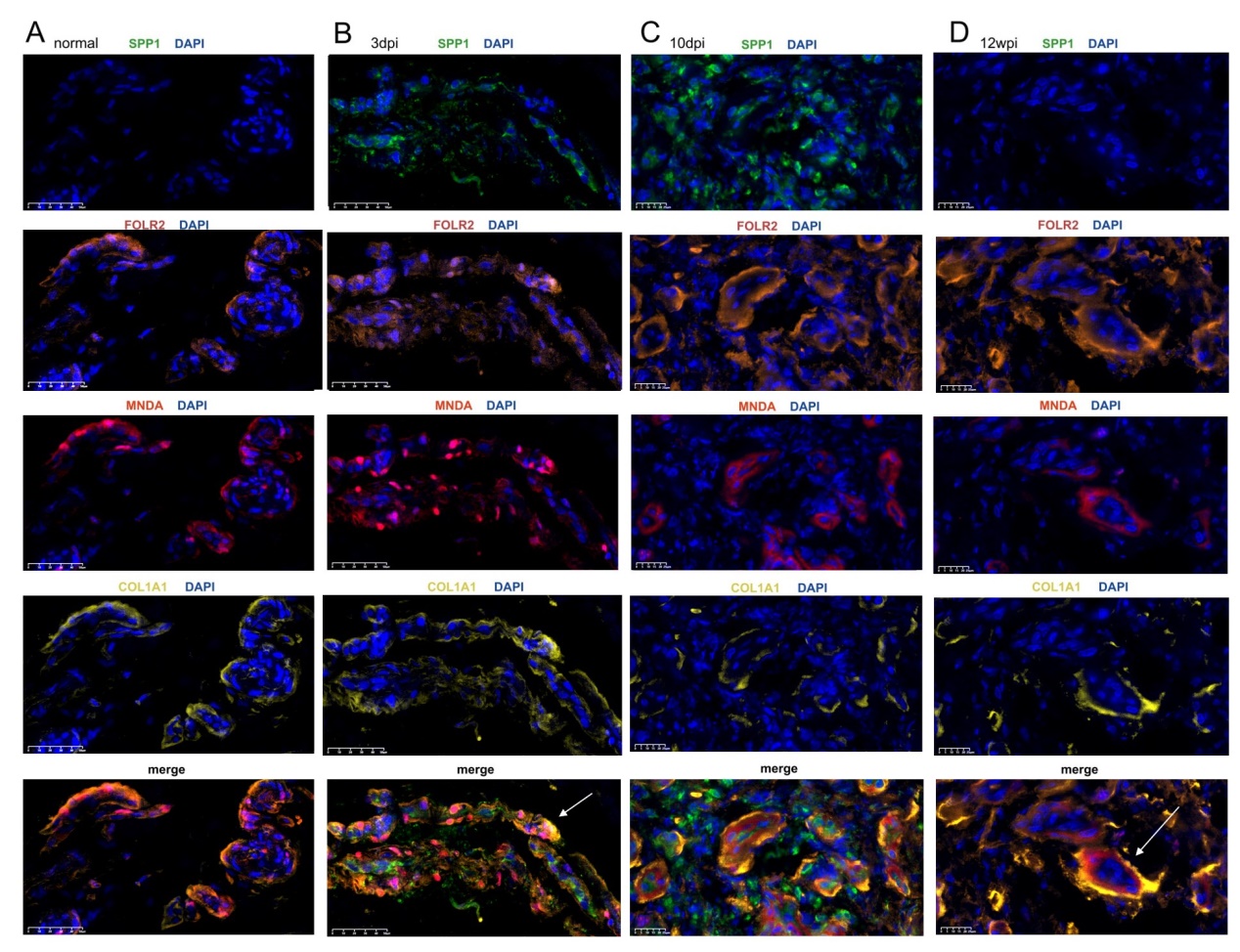


**Supplementary Figure 8. The immunofluorescence images of different time points.**

(**A**) The polychromatic immunofluorescence for *SPP1*, *FOLR2*, *MNDA* (the marker to monocytes) and *COL1A1* showed FOLR2^+^MNDA^+^ cells (white arrows) existed in human normal peritendinous tissue. *SPP1* (green) expression was low in normal peritendinous tissue. *SPP1* (green), *FOLR2* (orange), *MNDA* (red), *COL1A1* (yellow), *DAP*I (blue), scale bars 50μm.

(**B**) The polychromatic immunofluorescence for *SPP1*, *FOLR2*, *MNDA* (the marker to monocytes) and *COL1A1* showed SPP1^+^MNDA^+^cells and FOLR2^+^MNDA^+^cells (white arrows) existed around collagen at 3 dpi stage. White arrows FOLR2^+^MNDA^+^cells. *SPP1* (green), *FOLR2* (orange), *MNDA* (red), *COL1A1* (yellow), *DAPI* (blue), scale bars 50μm.

(**C**) The polychromatic immunofluorescence for *SPP1*, *FOLR2*, *MNDA* (the marker to monocytes) and *COL1A1* showed SPP1^+^MNDA^+^cells (*SPP1* and *MNDA* colocalization) and FOLR2^+^MNDA^+^cells (*FOLR2* and *MNDA* colocalization) existed at 10 dpi stage. *SPP1* (green), *FOLR2* (orange), *MNDA* (red), *COL1A1* (yellow), *DAPI* (blue), scale bars 25μm.

(**D**) The polychromatic immunofluorescence for *SPP1*, *FOLR2*, *MNDA* (the marker to monocytes) and *COL1A1* showed FOLR2^+^MNDA^+^cells (*FOLR2* and *MNDA* colocalization) existed at 12 wpi stage. White arrows FOLR2^+^MNDA^+^cells. *SPP1*(green), *FOLR2* (orange), *MNDA* (red), *COL1A1* (yellow), *DAPI* (blue), scale bars 25μm.


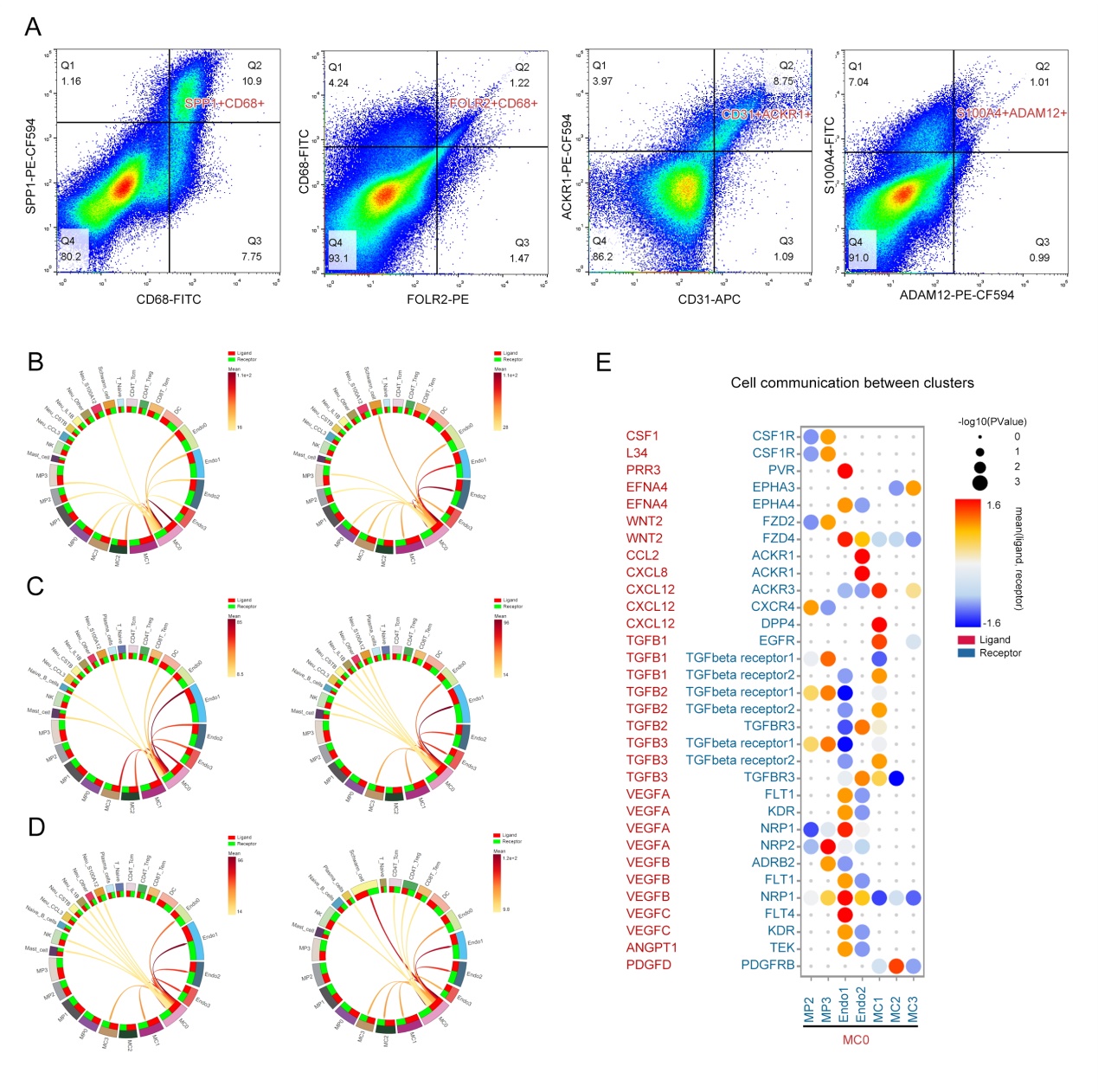


**Supplementary Figure 9. Flow cytometry and cellular interactions.**

(**A**) Flow cytometry: gating strategy for identifying human SPP1^+^macrophages, FOLR2^+^macrophages, ACKR1^+^Ecs and ADAM12^+^fibroblasts in 10 dpi tissue.

(**B**) Circosplot of the interactions between MC0 and other clusters at normal peritendinous tissue (Red, high. Yellow, low). Ligand, red. Receptor, green.

(**C**) Circosplot of the interactions between MC0 and other clusters at 3 dpi (Red, high. Yellow, low). Ligand, red. Receptor, green.

(**D**) Circosplot of the interactions between MC0 and other clusters at 12 wpi (Red, high. Yellow, low). Ligand, red. Receptor, green.

(**E**) Dotplot of ligand-receptor interactions between MC0 and ENDO1, ENDO2, MP2, MP3, MC1, MC2, MC3. Size of circle indicated P value (Big, high. Small, low). Color of circle indicated the mean of ligand and receptor expression levels in interacting clusters Left, ligands (red), receptors (blue). Left, receptors (red). Bottom, clusters.
